# Exploring generative AI in higher education: a RAG system to enhance student engagement with scientific literature

**DOI:** 10.3389/fpsyg.2024.1474892

**Published:** 2024-10-11

**Authors:** Dominik Thüs, Sarah Malone, Roland Brünken

**Affiliations:** Department of Education, Saarland University, Saarbrücken, Germany

**Keywords:** AI in education, technology acceptance model, scientific text comprehension, higher education, AI-powered learning environment, self-efficacy, ChatGPT

## Abstract

**Introduction:**

This study explores the implementation and evaluation of OwlMentor, an AI-powered learning environment designed to assist university students in comprehending scientific texts. OwlMentor was developed participatorily and then integrated into a course, with development and evaluation taking place over two semesters. It offers features like document-based chats, automatic question generation, and quiz creation.

**Methods:**

We used the Technology Acceptance Model to assess system acceptance, examined learning outcomes, and explored the influence of general self-efficacy on system acceptance and OwlMentor use.

**Results:**

The results indicated complex relationships between perceived ease of use, perceived usefulness, and actual use, suggesting the need for more dynamic models of system acceptance. Although no direct correlation between OwlMentor use and learning gains was found, descriptive results indicated higher gains among users compared to non-users. Additionally, general self-efficacy was strongly related to perceived usefulness, intention to use, and actual use of the system.

**Discussion:**

These findings highlight the importance of aligning AI tools with students’ needs and existing learning strategies to maximize their educational benefits.

## 1 Introduction

In many university courses, for example, in the social sciences, students are expected to study academic texts, such as primary research literature or research reviews in preparation for seminar sessions or exams. These papers, primarily written for seasoned scientists rather than learners, demand a robust level of scientific literacy. This literacy is essential for effectively connecting the central arguments, scientific methods, and data presented, thereby enabling readers to draw accurate and meaningful conclusions. Scientific literacy, as defined by the Program for International Student Assessment (PISA), is “The capacity to use scientific knowledge, to identify questions and to draw evidence-based conclusions in order to understand and help make decisions about the natural world and the changes made to it through human activity” (Organisation for Economic Co-operation and Development (OECD), 2003, p. 15). It involves understanding basic facts, concepts and processes, methods of scientific research, and the connections between science, technology, and society (Goldman and Bisanz, 2002). It cannot be assumed that all students possess sufficient scientific literacy to understand scientific texts (Sason et al., 2020), and compared to textbooks, engaging with academic papers can be particularly challenging for students. Given these challenges, new technologies such as generative AI hold great promise for improving students’ ability to engage with complex scientific literature. AI-powered tools have the potential to provide personalized, adaptive support that helps students navigate difficult concepts and connect new information to prior knowledge. However, as with previous technological advances, such as the introduction of computers in education, there are also risks. Relying too much on AI can bypass critical thinking processes and even introduce misinformation or bias. It is therefore crucial to identify the conditions under which AI tools can truly enhance learning. Research needs to be conducted on how these tools can be effectively integrated into educational environments while ensuring they are based on proven pedagogical strategies to support meaningful learning experiences. One promising approach is to combine generative AI with course-related knowledge bases that provide students with customized support for their academic needs. In the current study, we developed and evaluated an AI-based learning application, OwlMentor, designed to assist students in comprehending scientific texts. We conducted a longitudinal study to not only assess whether using OwlMentor positively affects students’ learning outcomes but also to determine the extent to which students voluntarily engage with the platform throughout various learning phases. Additionally, we examined whether this engagement could be explained by the Technology Acceptance Model (Davis, 1989), or if this theoretical model requires further expansion to accommodate changes in students’ engagement as they become more proficient in reading scientific texts over the semester.

### 1.1 Scientific text comprehension

Basic text comprehension involves creating preliminary mental representations during reading and refining them by comparing with existing knowledge. Coherence is achieved by resolving inconsistencies between prior knowledge and new information, eliminating contradictions, and connecting text elements and prior knowledge to form a coherent overall representation (Kintsch, 1998; Gernsbacher and Kaschak, 2013). Reading scientific texts is an interactive process where students engage with scientists’ ideas and arguments. It requires higher engagement than basic text comprehension, as readers must integrate complex information, critically evaluate the validity of arguments, understand methods, and apply theoretical knowledge. New students and novices often struggle with scientific texts due to unfamiliarity with the discipline’s structure and jargon (Goldman and Bisanz, 2002).

When students engage in science text reading, they often encounter significant challenges (Sason et al., 2020) related to their underdeveloped scientific literacy, including:

- Phenomenon Identification: The ability to correctly recognize and understand the main concepts, topics, or central scientific events discussed in a text. Obstacles include insufficient prior knowledge, complex presentations, or information overload (McNamara and Kintsch, 1996; O'Reilly and McNamara, 2007).
- Scientific Explanation: Being able to explain and understand scientific concepts in texts, requiring an understanding of complex relationships, familiarity with scientific jargon, the ability to critically analyze texts and the application of theoretical knowledge (Cromley et al., 2010; Norris and Phillips, 2003).
- Evidence Utilization: The understanding, interpretation, and evaluation of data to support or disprove conclusions. A Lack of statistical knowledge, for example, can lead to misunderstandings or ignoring crucial information. Studies highlight students’ difficulties in evaluating and integrating multiple sources of scientific evidence (Chinn and Brewer, 1998; Duncan et al., 2018).

These challenges underscore the need for strategies to enhance students’ understanding and engagement with scientific literature. Effective reading comprehension, especially for scientific texts, is supported by meta-cognitive strategies such as inferring unstated meanings, synthesizing information for cohesive understanding, and linking new information to prior knowledge. Effective methods for promoting text comprehension include:

- Self-questioning: Monitors and guides reading comprehension, aiding in identifying phenomena and encouraging active engagement and seeking clarification when needed (Gunn, 2008; Joseph and Ross, 2018; King, 1994).
- Linking new information to prior knowledge: Helps students understand complex relationships and scientific concepts by creating a familiar framework for new information (Kendeou and Van Den Broek, 2005, 2007; Sason et al., 2020).
- Summarizing key passages: Consolidates understanding by reinforcing main ideas and ensuring critical information is retained (Cromley et al., 2010).
- Self-explanation techniques: Students explain the material to themselves, deepening engagement with content by requiring them to process and articulate their understanding of scientific concepts (Chi et al., 1989; Chi et al., 1994).

Research has shown the effectiveness of these strategies. Gunn (2008) found that structured questioning, especially with high domain knowledge, enhances text memory and learning. Joseph and Ross (2018) demonstrated that self-questioning techniques tailored for middle school students with learning disabilities improve comprehension by generating questions before, during, and after reading. King (1994) showed that generating self-questions, particularly those linking new material with prior knowledge, promotes deeper knowledge construction and enhances learning outcomes. Kendeou and van den Broek (2005) found that misconceptions significantly influence text comprehension as they affect memory representation of the text. Their 2007 study showed that prior knowledge and text structure interact to influence cognitive processes during reading, with explicit disconfirmation of misconceptions improving comprehension. Sason et al. (2020) demonstrated that comprehension of science texts improves significantly when students learn to ask questions that connect to the text. Cromley et al. (2010) found that effective reading strategies, such as summarization, are strongly associated with improved comprehension and academic achievement in science, suggesting that supporting students in summarizing key information can significantly enhance their understanding and retention of scientific concepts. Chi et al. (1989) found that good students generate detailed self-explanations, which refine and expand their understanding, leading to better problem-solving skills and independent knowledge. Chi et al. (1994) demonstrated that self-explanation promotes deeper understanding and integration of new information, as students who self-explained while reading showed greater knowledge gains and constructed more accurate mental models.

However, applying strategies such as self-questioning, linking to prior knowledge, summarizing, and self-explaining is challenging for learners, requiring significant (meta-) cognitive and motivational resources. This can be a barrier to their successful application. Generative artificial intelligence (GAI) and large language models (LLMs) can support these methods by providing interactive engagement, generating practice questions, and offering feedback. These technologies have the potential to enhance students’ comprehension of academic texts through targeted support and practice, showing promise for university teaching (Kasneci et al., 2023).

### 1.2 Generative AI assistants to support text comprehension

To effectively develop generative AI assistants that aid students in understanding scientific texts required in university courses, three key theoretical considerations arise. First, it is crucial to translate the capabilities of GAI into pedagogical functionalities of a learning application that can foster meta-cognitive strategies beneficial for comprehending scientific texts. Second, measures must be implemented to ensure that the system produces outputs that are both accurate and relevant. Third, it is important to identify factors that influence whether students will engage actively with the system over an extended period, such as the duration of a semester. In the following sections, we will delve into the theoretical underpinnings of these three aspects.

GAI can be defined as Artificial Intelligence (AI) that generates new data or outputs, using machine learning (Gimpel et al., 2023). LLMs in particular offer a wide range of promising applications in the education sector (Kasneci et al., 2023). They are trained on a large corpus of data to process and generate natural language text (Gimpel et al., 2023). Natural language processing (NLP) aims to enable computers to understand and process human language. Significant progress in this field has been achieved through the introduction of Transformer models (Vaswani et al., 2017), such as BERT and GPT that allow the context of a word to be analyzed in relation to all other words in the text, resulting in improved processing speed and accuracy. Since ChatGPT ‘s launch in 2022, numerous studies have taken a closer look at the benefits and challenges of LLMs and conversational AI in education, such as GPT-3.5 and GPT-4 (Cooper, 2023; Herft, 2023; Kasneci et al., 2023; Pavlik, 2023; Qadir, 2023; Sallam, 2023; Zhai, 2022). For instance, LLMs have been employed to create educational content, including quizzes and flashcards (Bhat et al., 2022; Dijkstra et al., 2022; Gabajiwala et al., 2022), function as pedagogical agents or conversation partners (Abdelghani et al., 2022; Bao, 2019; El Shazly, 2021; Ji et al., 2023), and serve as tools for providing feedback (Jeon, 2021). Based on this research, written initial guidelines as well as recommendations on how to possibly integrate them into educational settings were developed (Gimpel et al., 2023; Kasneci et al., 2023; Mollick and Mollick, 2022). Besides ChatGPT’s interface, users can access models like GPT 3.5 and GPT 4 to build their own applications.

In the field of educational applications, it seems particularly promising that conversational AI assistants can be developed that interact with learners in a human-like way and help them for example, with understanding given scientific texts. A recent meta-analysis (Wu and Yu, 2023) suggests that the use of conversational AI can increase students’ performance, motivation, and self-efficacy and reduce anxiety, especially at the university level. Other literature reviews state that conversational AI enhances student skills and motivation (Wollny et al., 2021), significantly impact learning achievement and satisfaction (Kuhail et al., 2022) and facilitate language learning (Huang et al., 2022). Liang et al., 2023 found that GAI interaction can boost self-efficacy and cognitive engagement, both serving as mediators for learning achievement, with GAI interaction also having a direct effect on learning achievement. Although it has not yet been explicitly explored, conversational AI assistance presents a wealth of opportunities for enhancing students’ comprehension of scientific texts. Leveraging the advanced capabilities of LLMs, these AI systems excel at inferring meanings, synthesizing information, and connecting concepts within selected scientific texts. Such proficiency suggests that conversational AI provided through pedagogically informed applications could serve as an effective mentor, potentially surpassing student capabilities in these complex cognitive tasks. When considering the use of LLMs in this capacity, it must be taken into account that AI systems are only as good as their training data and are associated with biases and misinformation (Alkaissi and McFarlane, 2023; Qadir, 2023), limitations in the scope of knowledge, lack of interpretability (Kasneci et al., 2023), the exacerbation of ethical issues, unreliability, toxicity (Zhuo et al., 2023), and risks of technical dependence and misuse (Alshater, 2022; Kasneci et al., 2023). For students new to a topic, these risks are particularly pertinent given that for them it is challenging to verify an LLM’s accuracy. Recent studies indicate that the accuracy of ChatGPT responses is around 60% (Kung et al., 2023) and that 52% of the software development responses contained inaccuracies (Kabir et al., 2023). However, it is important to emphasize that such analyses are snapshots and that LLMs such as ChatGPT are continuously evolving. Moreover, there are currently at least two robust methods available to refine LLM responses to prevent learners’ misinformation: 1. fine-tuning a pre-trained LLM with one’s own data set, which is very cost-and time-intensive, or 2. sending additional information with the initial user prompt (e.g., chain of thought techniques, zero/few-shot prompting or in-context learning; Zhao et al., 2023), which can be used in the short term and with manageable effort. In-Context Learning enhances contextual understanding and aids in mitigating errors like hallucinations, where the LLM generates seemingly credible but inaccurate information (Alkaissi and McFarlane, 2023). In-Context Learning offers a practical option, especially for teachers who are not experts in computer science. This method makes it also possible to make short-term and minor adjustments to teaching materials (such as the selection of scientific texts) with relatively little effort.

A widely used way to apply contextual learning to an LLM is retrieval augmented generation (RAG), where hallucinations can be reduced by using information retrieval methods to provide additional context to a prompt (Shen et al., 2023). A RAG system involves searching and retrieving documents that semantically match a query and then passing these documents to an LLM. Usually, the documents are retrieved from the database based on the user request and then transmitted to the LLM via a prompt. Such RAG systems aim to reduce the problem of hallucinations, link references to generated responses or remove the need for annotating documents with meta-data (Barnett et al., 2024). It could be shown that RAG Systems can substantially increase accuracy in some cases (94% improvement over situations where no context is provided) but can still be misled if prompts directly contradict the previously trained understanding of the model (Feldman et al., 2023, 2024). RAG systems provide a great opportunity to equip LLMs with specific knowledge. Especially for educational scenarios where specific scientific literature is provided, RAG-based applications could be valuable to support students.

### 1.3 Technology acceptance model

Even the best learning applications cannot promote learning if the learners use it minimally or fail to utilize all its helpful features. A theory frequently employed to explain how new software or information technologies are adopted by learners is the Technology Acceptance Model (*TAM*; Davis et al., 1989, Venkatesh and Davis, 1996). The TAM was developed to explain and predict how users accept and implement new technological tools. Over the years, TAM has been frequently studied and extended (for an overview, see Chuttur, 2009; Yousafzai et al., 2007a). It focuses on four central constructs: Perceived Ease of Use, Perceived Usefulness, Intention to Use, and Actual System Use. Perceived Ease of Use evaluates whether potential users perceive the technology as easy to operate. Technologies considered easy to use are more likely to be adopted, as they reduce the effort required for learning and using the system (Venkatesh and Davis, 2000). Perceived Usefulness refers to the belief that the technology will improve one’s performance (Opoku and Enu-Kwesi, 2019). Intention to Use describes the extent to which a person has the behavioral Intention to Use the technology. Actual System Use is the actual behavior of users, indicating how often and to what extent the technology is used. According to the extended TAM (Venkatesh and Davis, 1996), both Perceived Ease of Use and Perceived Usefulness directly influence Intention to Use, which in turn is a strong predictor of Actual System Use. External factors such as system experience, educational level, digital Self-Efficacy and age can influence Perceived Ease of Use and Perceived Usefulness. However, there is no consensus on these external factors, as different studies have identified varying influencing variables (Chuttur, 2009; Yousafzai et al., 2007a).

Over the years, the TAM has been frequently studied and extended (Chuttur, 2009; Yousafzai et al., 2007a). These studies show that Perceived Usefulness consistently emerges as a significant predictor of technology acceptance, while the influence of Perceived Ease of Use may vary or decrease over time. For example, Davis et al. (1989) observed that the influence of Perceived Ease of Use on behavioral Intention to Use tends to diminish as users become more familiar with a technology. Further research supports this, showing that the effect is more pronounced in the early stages of technology adoption, but becomes less evident over time (Adams et al., 1992; Chau, 1996; Gefen and Straub, 2000; Igbaria et al., 1996). Subramanian (1994) also found that Perceived Ease of Use has less influence on Actual System Use when the technology is inherently easy to use. The validity of the TAM was mainly confirmed by studies in which data were collected at a single point in time, usually shortly after introducing a new technology. However, Yousafzai et al.’s (2007b) meta-analysis shows a gap in understanding how these relationships change over longer periods. This analysis highlights the need for longitudinal research to capture the evolving nature of technology adoption, as many TAM studies have not considered longer-term changes or new variables that may become relevant as users continue to engage with the technology. Future TAM research should therefore take more longitudinal and dynamic approaches to understand better how users’ intentions evolve (Davis et al., 2024). Recent studies continue to support and expand upon the core constructs of TAM. Yu et al. (2024) demonstrated that Perceived Ease of Use and Perceived Usefulness are strong predictors of continued Intention to Use, with Perceived Usefulness being the strongest predictor. Zou and Huang (2023) highlighted that attitude towards using technology significantly mediates the effects of Perceived Usefulness and Perceived Ease of Use on Intention to Use. These results suggest that while the core constructs of the TAM are still relevant, the use of Perceived Ease of Use and Perceived Usefulness as predictors still needs to be validated in new contexts, including the use of ChatGPT and other LLMs in education.

### 1.4 OwlMentor: an AI-powered learning environment

Our AI-powered learning environment, OwlMentor, was developed using principles from User-Centered Design (UCD) to focus on usability and usefulness. UCD is an iterative process focusing on users’ needs at different design stages to ensure the final product meets their preferences (Norman and Draper, 1986). OwlMentor, named after our wise university mascot, includes features to enhance scientific text comprehension. It supports self-questioning through free chat sessions about course literature, allowing students to ask questions and receive immediate answers, fostering active engagement. OwlMentor also generates summaries of scientific texts, helping students understand key passages and main ideas. Additionally, it automatically creates multiple-choice questions from the text, enabling students to test their knowledge and practice regularly. This process involves deciding whether to keep or discard the questions, ensuring students´ active involvement. These questions can be compiled into quizzes, promoting a constructive learning environment where students generate outcomes beyond the provided information. At the end of a quiz, OwlMentor provides feedback for each question if requested, enhancing self-explanation by having students articulate why an answer is correct or incorrect. By offering these functions, OwlMentor has the potential to facilitate deeper engagement with course content and improve students’ ability to comprehend complex scientific texts.

OwlMentor was developed by the authors as part of the ‘Innovation Project OwlMentor,’ which was part of the ‘Digital Teaching Plug-in’ (DaTa-Pin) project funded by the Foundation for Innovation in Higher Education Teaching (Stiftung für Innovation in der Hochschullehre), granted to Saarland University. This project extended over two semesters and took place in two consecutive courses in a master’s program in Educational Technology. In the first semester, participatory prototype development was carried out with the involvement of the students, which was afterward revised and offered to the students during the second semester for evaluation. During the first semester, our focus was on the functionality and quality of the OwlMentor responses while also trying to design a good user experience. As part of the revision during the transition between semesters, we applied the results gained from participatory development with the students to enable a user experience that is both appealing and of high content quality. Given this background, our work can be understood as an exploratory study on the development and integration of AI-based applications in university teaching. The aim of the present study was to develop, implement, and evaluate the integration of OwlMentor into an existing university course, where students must prepare for the course lessons by reading and understanding specific scientific texts provided by the lecturer. Our goal is to improve text comprehension and reinforcement of course content by providing an AI-powered learning environment in which students can interact with a conversational AI and receive support in creating self-assessment questions and quizzes that they can practice and solve.

### 1.5 Research interest and hypothesis

Our research interest focuses on the use and impact of OwlMentor in a university course. We wanted to explore how students interact with this AI-based learning platform, focusing on their usage behavior, the benefits they derive from this usage, and the overall impact on their learning process. We utilized the TAM as a framework for system evaluation to predict the factors influencing students’ engagement with OwlMentor. The TAM posits that two central constructs, Perceived Ease of Use and Perceived Usefulness, directly influence the Intention to Use a technology, which subsequently predicts Actual System Use. Based on this model, we formulated the following hypotheses:

- H1: Perceived Ease of Use is positively related to Intention to Use.
- H2: Perceived Usefulness is positively related to Intention to Use.
- H3: Higher Intention to Use leads to higher Actual System Use.

In addition to evaluating the system’s acceptance, we also aimed to assess its effectiveness as an educational tool through the following hypothesis:

- H4: Higher Actual System Use leads to higher learning gains.

These hypotheses aim to capture the relationship between the design and usability factors of OwlMentor and investigate its acceptability and effectiveness as an educational tool. Our objective is not only to evaluate the practical impact of the OwlMentor but also to contribute to a broader understanding of how such technologies can be designed and implemented to improve educational outcomes. Additionally, we included a measure of general Self-Efficacy to explore its potential influence on students’ engagement with OwlMentor. General Self-Efficacy, defined by Bandura (1982), refers to an individual’s belief in their ability to succeed in various tasks and challenges. This concept is broader than digital Self-Efficacy, which focuses specifically on confidence in using technology. Research has shown that general Self-Efficacy is linked to improved motivation and learning strategies (Pintrich and De Groot, 1990). Given that previous studies have indicated a positive impact of generative AI on general Self-Efficacy (Liang et al., 2023; Wu and Yu, 2023), we aimed to investigate whether interacting with OwlMentor could enhance students’ overall confidence in managing the course demands. Based on these considerations, we have made an initial attempt to integrate the AI-based application OwlMentor into teaching, examining both system acceptance and effectiveness in terms of text comprehension and student engagement.

## 2 Materials and methods

### 2.1 Participants

The participants of the present study were international students on the Master’s degree program in Educational Technology (EduTech) at a German University. The students attended two consecutive courses in the Learning with Media module, Multimedia Learning I and Multimedia Learning II, which spanned two semesters. In the Multimedia Learning I course in the first semester, a prototype of the OwlMentor was presented and tested together with the students as part of a pilot study. In the second semester, the main study was carried out by implementing and evaluation a further developed version of the OwlMentor in the course. During the courses, OwlMentor was made available to the students for voluntary use in order to support them in their work with course-relevant scientific literature.

Students independently enrolled in the Multimedia Learning courses, which are part of a compulsory module within the master’s program, via the university’s internal registration system. Their consent to participate in the study was obtained after they had been introduced to OwlMentor. Apart from meeting the general enrollment requirements for the course, there were no other exclusion criteria. The course allowed a maximum of 25 EduTech students per cohort and, although students could register from their first semester, participation was recommended for those who were in their second or third semester.

The course participants were international students with an interdisciplinary background. All participants had at least a bachelor’s degree in either a computer science subject (e.g., computer science, data science), education (e.g., teaching degree) or psychology. In terms of their previous knowledge, they could therefore be described as rather heterogeneous. There were two native English speakers in both semesters and all others had at least B2 level (requirement for the EduTech program). In the pilot study (Multimedia Learning I course), the sample consisted of 17 students with an average age of *M* = 27.59 years (*SD* = 2.29) and a balanced gender distribution (female: *n* = 8, male: *n* = 9). Regarding their prior knowledge, the students stated in a self-assessment that they had on average moderate knowledge of multimedia learning (*n* = 8) and that some already had knowledge of multimedia formats (*n* = 3), multimedia principles (*n* = 3), and cognitive load theory (*n* = 3). In the main study (Multimedia Learning II course) the sample consisted of 16 of the former 17 students with an average age of *M* = 27.38 years (*SD* = 2.19) and a balanced gender distribution (female: *n* = 8, male: *n* = 8).

### 2.2 Multimedia learning courses

Both Multimedia Learning Courses were part of the module Learning with Media which has a duration of two semesters and is compulsory in the master program. The Multimedia Learning I course teaches the basics of learning with multimedia instructions, such as the Cognitive Theory of Multimedia Learning (Mayer, 2014), the Cognitive Load Theory (Sweller, 2011) and basic principles of multimedia learning such as the Multimedia Principle, Modality Principle, or the Redundancy Effect (Mayer and Fiorella, 2021). The seminar was held in summer term 2023 in a blended learning format, comprising eight synchronous classroom sessions, four asynchronous assignment sessions, a presentation session, and a final exam. During this seminar, the OwlMentor prototype was presented, tested, and evaluated. The Multimedia Learning II course teaches further principles of multimedia learning: Expertise-Reversal Principle, Split-Attention Principle, Worked Example Principle, Principles based on Social Cues and Emotional Design Principle (Mayer and Fiorella, 2021). This seminar was also held in a blended learning format in the winter term 2023/24. The course included six on-site content sessions and six asynchronous sessions for preparing scientific literature, held on alternating weeks. The first synchronous session was led by lecturers; subsequent sessions were prepared and conducted by student groups. Additionally, there were an organizational session, a mock exam session, and a final exam session. Students prepared chapters from the Handbook of Multimedia Learning (Mayer and Fiorella, 2021) and relevant research articles. At the seminar’s start, OwlMentor and its functions were introduced, with anonymous access, a manual, and instructional videos provided. Throughout the seminar, the advanced OwlMentor version was freely available for dialogue, question generation, practice quizzes, and AI-generated feedback.

### 2.3 Pilot study (first semester)

The main aim of our pilot study was to test the technical functionality of the OwlMentor and the quality of its AI-generated answers and questions. We used the System-Usability-Scale (SUS; Brooke, 1996), a 10-item questionnaire with a reported internal consistency of Cronbach’s alpha = 0.91 (Bangor et al., 2009). In this early version, the dialog function and question generation function were tested for one course topic. At first students were introduced to the Cognitive Theory of Multimedia Learning (Mayer, 2014) and answered nine questions of different educational objective taxonomy levels (remembering, understanding, applying, evaluating; Bloom, 1956). Example questions include “Define the term ‘multimedia learning’ according to Mayer and Fiorella’s handbook,” “What Are the Cognitive Processes Involved in Active Learning (SOI model)?” and “Explain the limited capacity assumption of the cognitive theory of multimedia learning.” Then the students, divided into groups, asked these questions to the OwlMentor and rated its responses from 1 (very good) to 6 (unsatisfactory). To assess the automatic question generation function, students created six questions per group using selected text passages. They rated the generated questions on difficulty (1 = low, 5 = high) and usefulness (1 = low, 5 = high). Examples of generated questions are “Which of the following is NOT a type of Cognitive Load? Options: A: Intrinsic Load, B: Extrinsic Load, C: Germane Load, D: Emotional Load” and “Which of the following is NOT one of the three cognitive processes essential for active learning according to the SOI model? Options: A: Selecting relevant material, B: Organizing selected material, C: Integrating selected material with existing knowledge, D: Evaluating the effectiveness of the learning material.”

Students rated the response quality with a mean value of 2.19 (*SD* = 0.99), with ratings for Remembering (*M* = 2.29, *SD* = 0.83), Understanding (*M* = 1.88, *SD* = 0.33), Applying (*M* = 2.8, *SD* = 2.21), and Evaluation (*M* = 1.63, *SD* = 0.95). The difficulty of generated questions had a mean value of 1.92 (*SD* = 0.26) and usefulness a mean value of 3.29 (*SD* = 0.46). The students rated the usability of the prototype on the SUS with a value of 56.25, corresponding to the adjective “ok” (Bangor et al., 2009). A short user feedback questionnaire (*n* = 5) and discussions with the students revealed that they found it easy to chat and generate questions with OwlMentor but had mixed opinions about its understanding of user queries, clarity of responses, and overall satisfaction. Specifically, they noted that the AI’s responses were sometimes too long and not sufficiently precise, making it challenging to find the exact information they needed. They also mentioned that the user interface was not intuitive and that response times were slow, affecting their overall experience. Moreover, students expressed a desire for additional functionalities, such as the ability to upload or use PDF’s in the application. Overall, they believed that while OwlMentor supported their understanding of the course literature, there was room for improvement. Based on their suggestions, we implemented several modifications to OwlMentor, as detailed in Section 2.5.3.

### 2.4 Main study design

The aim of the main study was to integrate and evaluate the extended version of OwlMentor in the Multimedia Learning II course. It was an exploratory study in a pre−/posttest design. Due to the small number of participants and to ensure that all students had equal opportunities in the course, no control group design was used. The dependent variables for the evaluation of OwlMentor were students’ learning gains (difference pre/posttest), usability in terms of the TAM model (Perceived Ease of Use, Perceived Usefulness, Intention to Use, Actual System Use), and an expert assessment of OwlMentor’s response quality with two independent raters. The log data was analyzed to assess the Actual System Use of the application. The participants’ Self-Efficacy was recorded as an additional variable and the interactions with the OwlMentor were also analyzed qualitatively to get an impression of how the students engaged with the application.

### 2.5 OwlMentor

OwlMentor is an AI-powered web application designed to assist students in comprehending scientific texts required for their courses. This section provides an overview of OwlMentor’s structure, development, core functionalities, and technical implementation, addressing key aspects of its design.

#### 2.5.1 User interface and core functionalities

The user interface comprises four main sections—Dashboard, Chat, Questions, and Quiz—accessible through a navigation bar.

**Figure 1:**
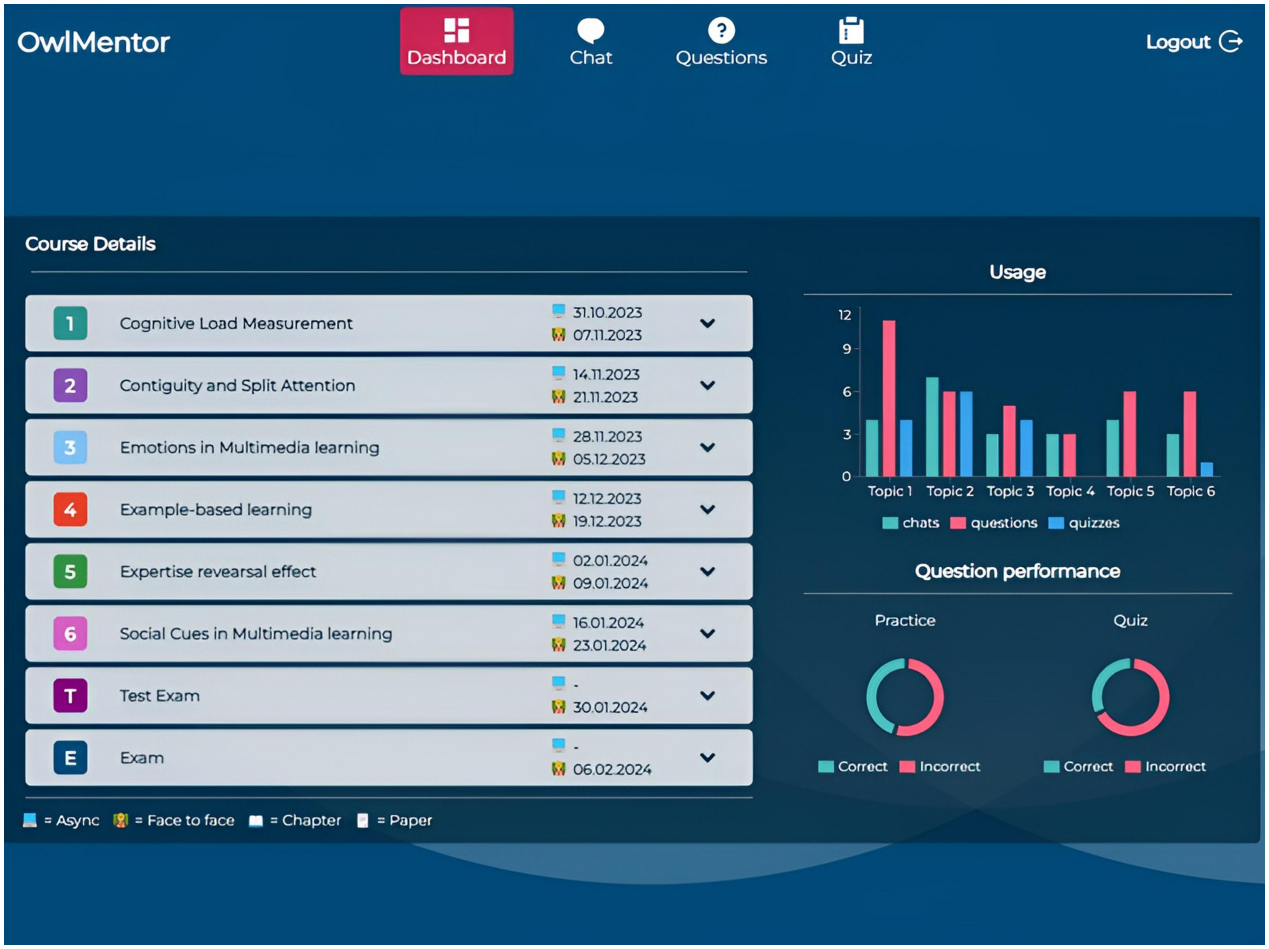
Application screenshot dashboard.

**Figure 2:**
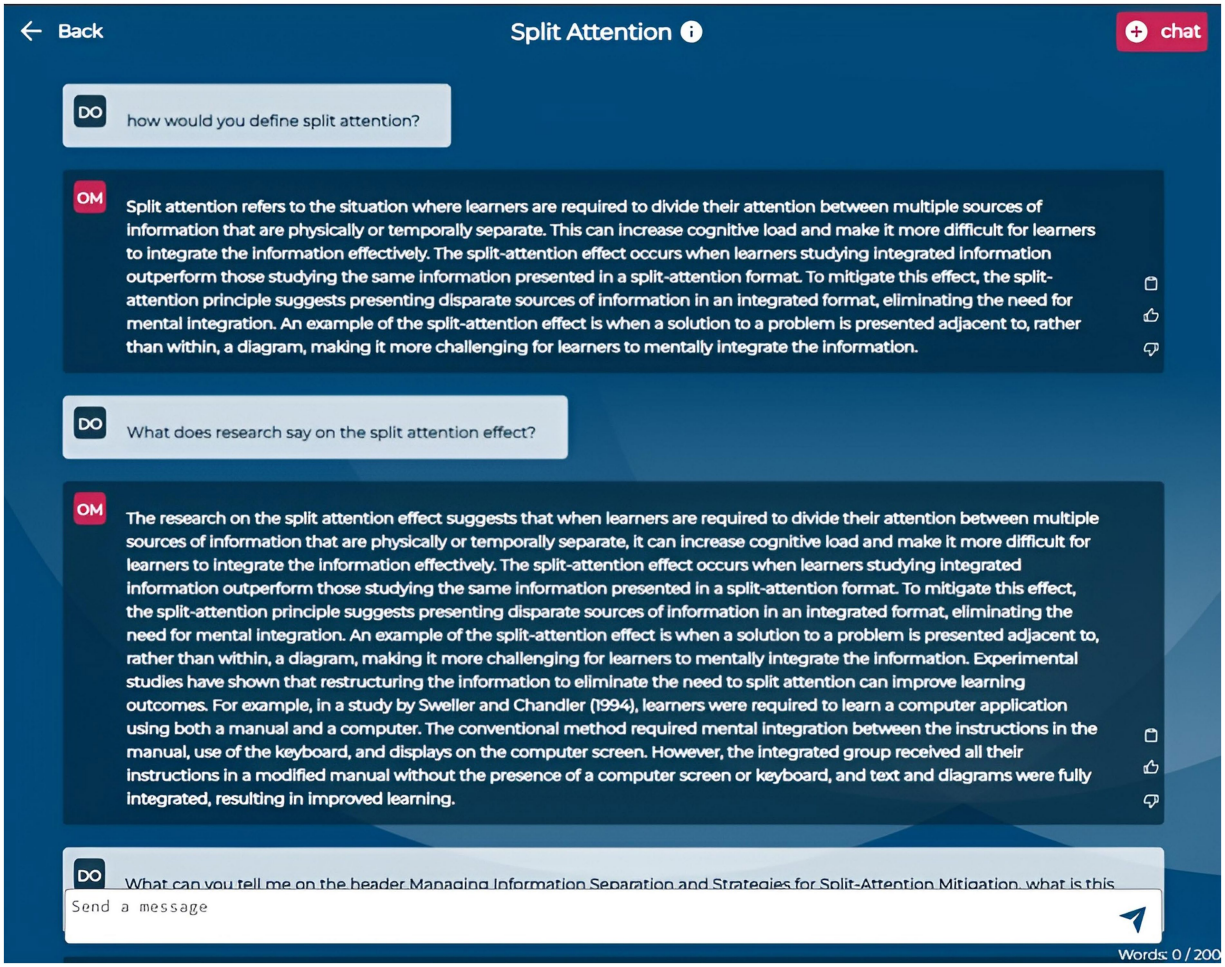
Application screenshot dialog function.

The Dashboard (Figure 1) provides an overview of course details, including topics, session dates, and linked required literature. It also displays usage statistics such as the number of chats initiated, questions generated, and quizzes taken, allowing students to monitor their progress. In the Chat section (Figure 2), students can continue an existing conversation or start a new one about specific course documents. The chat interface allows them to engage in free-form dialogues with OwlMentor. This document-based chat enables students to ask questions, seek clarifications, and explore concepts in depth. By retrieving relevant information from the selected document, OwlMentor provides accurate, context-specific responses based on students’ queries - such as summarizing content, explaining concepts, or defining terms - which may assist them in understanding the material.

**Figure 3:**
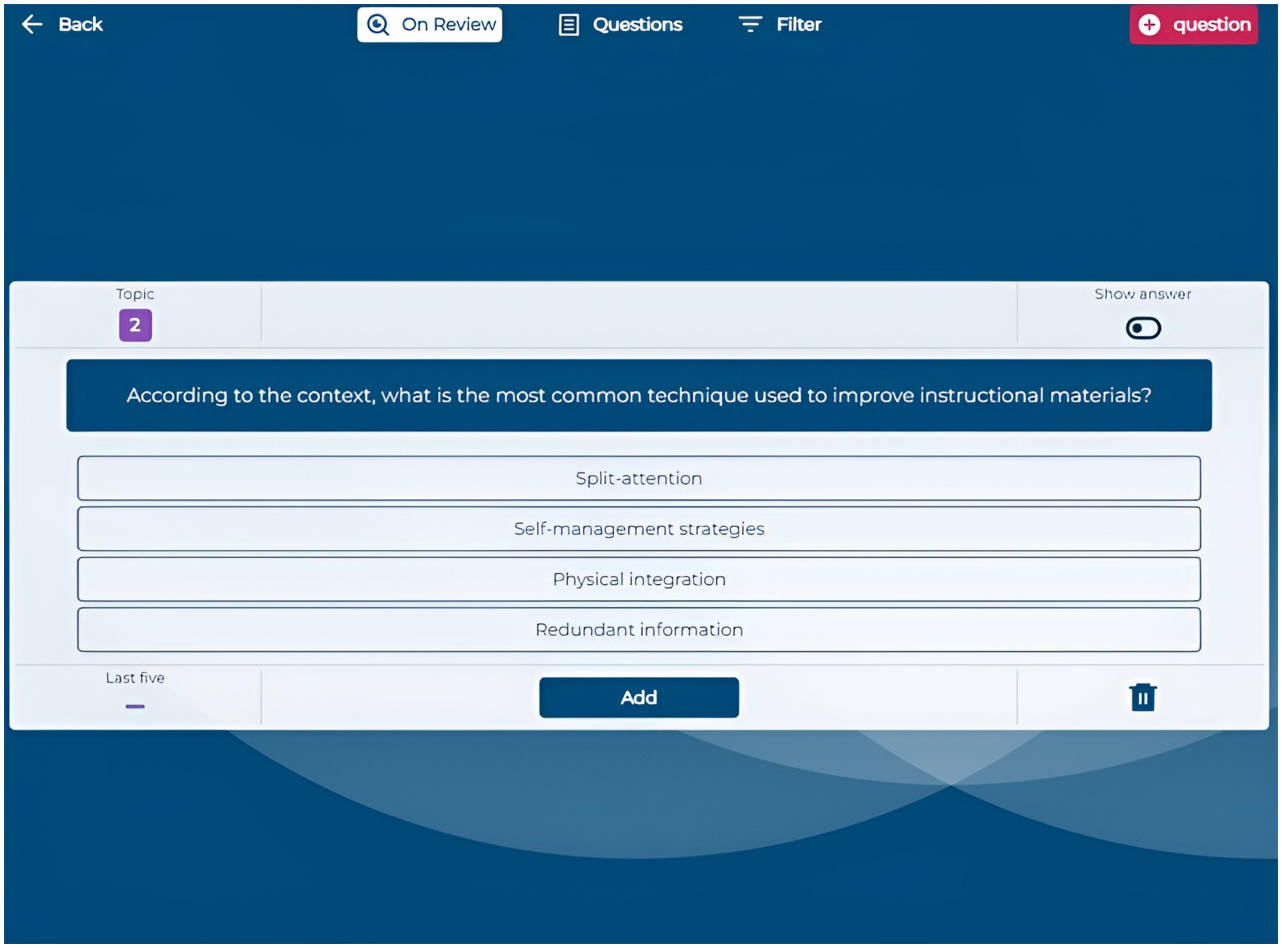
Application screenshot on review area.

**Figure 4:**
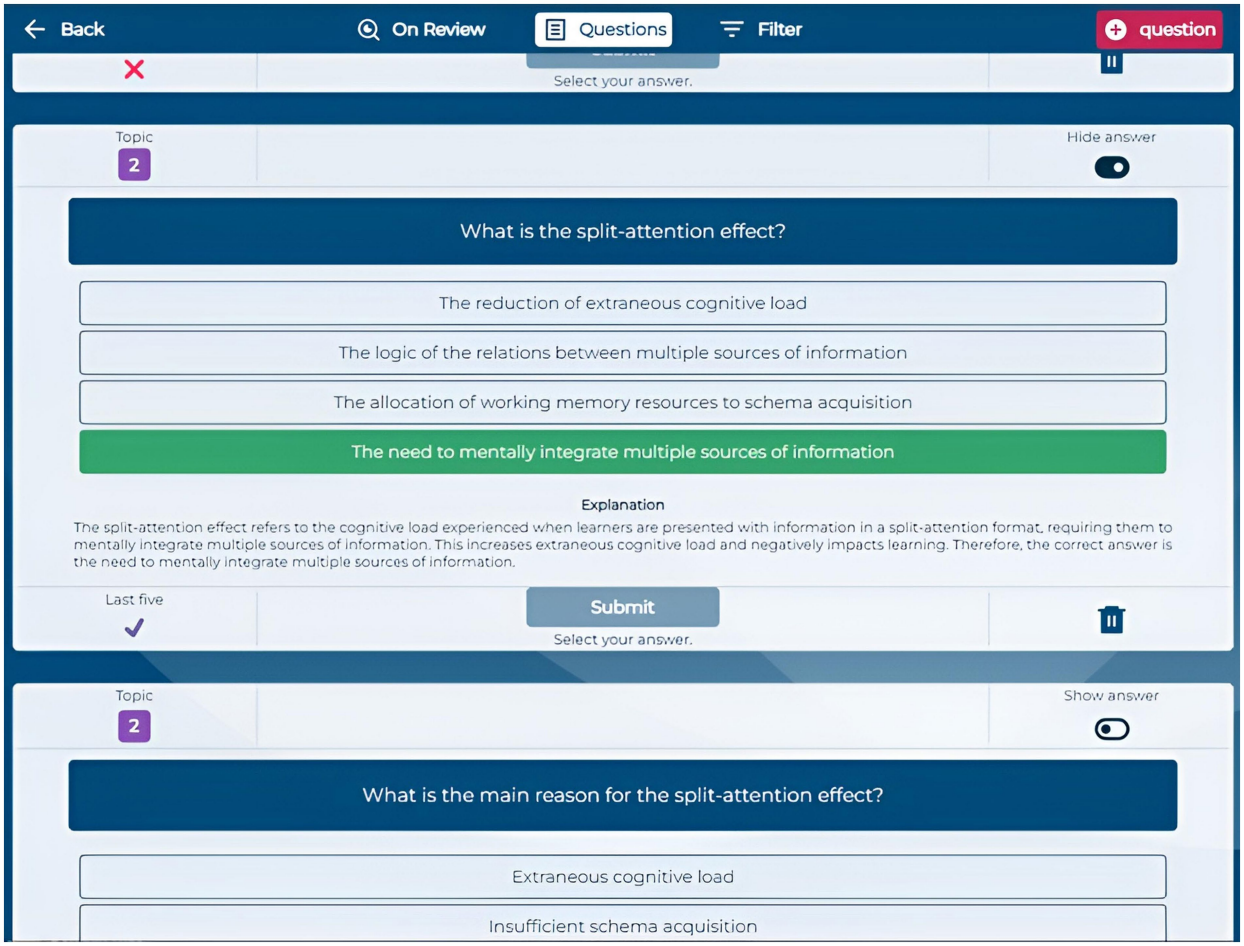
Application screenshot question generation.

The Questions section enables students to generate multiple-choice questions automatically by providing text from course documents. They can input specific text excerpts, upload highlighted PDFs, or let the system select random sections. Generated questions appear in an “On Review” section, where students review and validate each question before adding it to their personal question bank. This automatic question generation prompts students to actively engage with the content by critically assessing the quality and relevance of each generated question and its answers. By evaluating correctness and clarity, students deepen their understanding and reinforce key concepts (Figures 3, 4).

**Figure 5:**
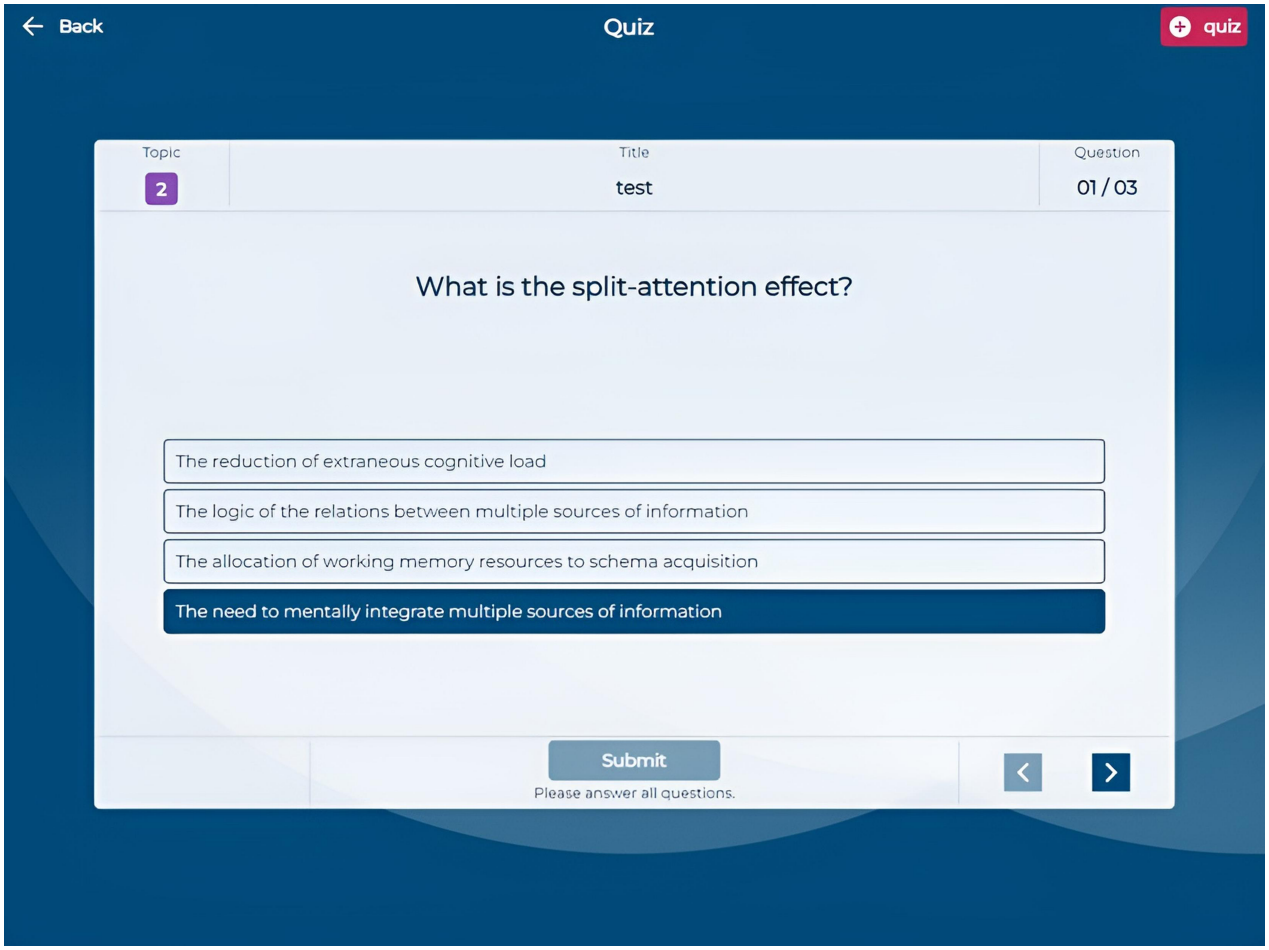
Application screenshot quiz.

**Figure 6:**
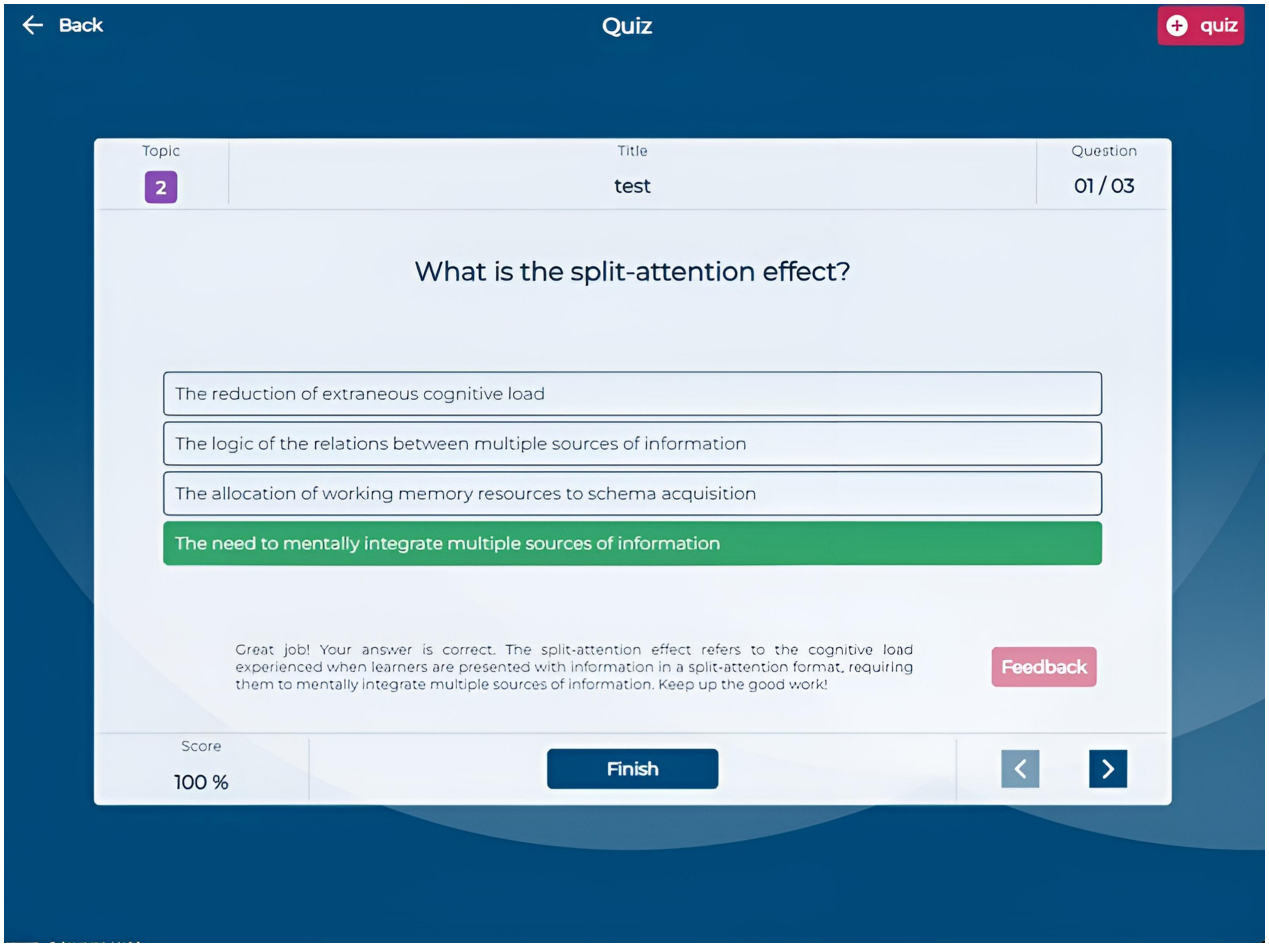
Application screenshot quiz feedback.

The Quiz section allows students to create custom quizzes by selecting a title, the number of questions (3, 6, or 9), and specific questions from their question bank. The quiz interface (Figure 5) presents questions sequentially, enabling navigation and answer submission. After completing a quiz, students receive immediate feedback indicating which questions they answered correctly or incorrectly, along with a brief motivational message based on their performance. They can request detailed, AI-generated feedback on each question to enhance their comprehension (Figure 6). This quiz creation and feedback functionality allows students to assess their knowledge and understand the reasoning behind each answer, as the AI provides explanations that clarify misunderstandings and reinforce learning.

#### 2.5.2 Development phases

OwlMentor was developed iteratively over three main phases, incorporating user feedback and technical enhancements. Prior to submitting the proposal for the OwlMentor teaching and research project, the authors conducted a Proof of Concept (Version 1) phase to assess the feasibility of integrating a RAG system. By developing a basic RAG architecture with a simple chat interface, they confirmed its viability and proceeded with further development. During the Prototype Testing with Students (Version 2) phase, a pilot study was conducted (Section 2.3) where students tested the prototype and provided feedback on usability, response quality, and satisfaction. Based on their input, the following modifications were made:

- User Interface Enhancements: Students reported that the interface was unintuitive and that response times were slow, affecting usability as reflected in the SUS scores. The interface was redesigned for better clarity and navigation, incorporating a cleaner layout and more intuitive controls. Performance was optimized by implementing streaming responses in the chat function, reducing perceived latency.
- Improved Response Quality: The AI’s responses were too long and imprecise because the language model searched across all documents that have similar wording (e.g., multimedia learning, cognitive load), making it hard to find correct contexts. To address this, the retrieval strategy was refined to focus on specific documents using logical routed retrieval and adjusted system prompts to produce shorter, more precise answers.
- Expanded Functionality: Students requested the ability to generate questions from PDFs, as the initial version only allowed copying and pasting text. A feature was added enabling users to upload highlighted PDFs for automatic question generation and an option to create random questions from selected documents was introduced.
- Additional Engagement Features: To enhance engagement, a quiz function was introduced allowing students to compile questions into custom quizzes where correct answers were not immediately revealed, increasing the challenge. We also added the option to receive elaborative feedback on quiz questions to support deeper learning.

In the Final Version (Version 3), these improvements were integrated, offering enhanced retrieval processes, detailed feedback options, and a streamlined user experience for the main study. The user interface designs for all three versions of OwlMentor can be viewed in Appendix 6.

#### 2.5.3 Technical implementation

OwlMentor’s AI functionalities rely on OpenAI’s GPT-3.5 Turbo model (version 0613) to provide accurate, context-specific responses, generate questions, and offer feedback. Each core function—dialogue, question generation, and feedback—sends tailored requests to the OpenAI API, optimizing performance and precision. These requests are guided by specific system prompt templates (Appendix C) that instruct different instances of the LLM (e.g., Strategy-, Response Model) on how to process user queries and respond appropriately based on the context.

The backend architecture of OwlMentor is built with Python and FastAPI, using MongoDB for data storage, and the Annoy library to construct a vector database that allows efficient similarity searches for document retrieval. A vector database stores vector representations (or embeddings) of document chunks, enabling fast searches based on the similarity between user queries and the stored vectors.

**Figure 7:**
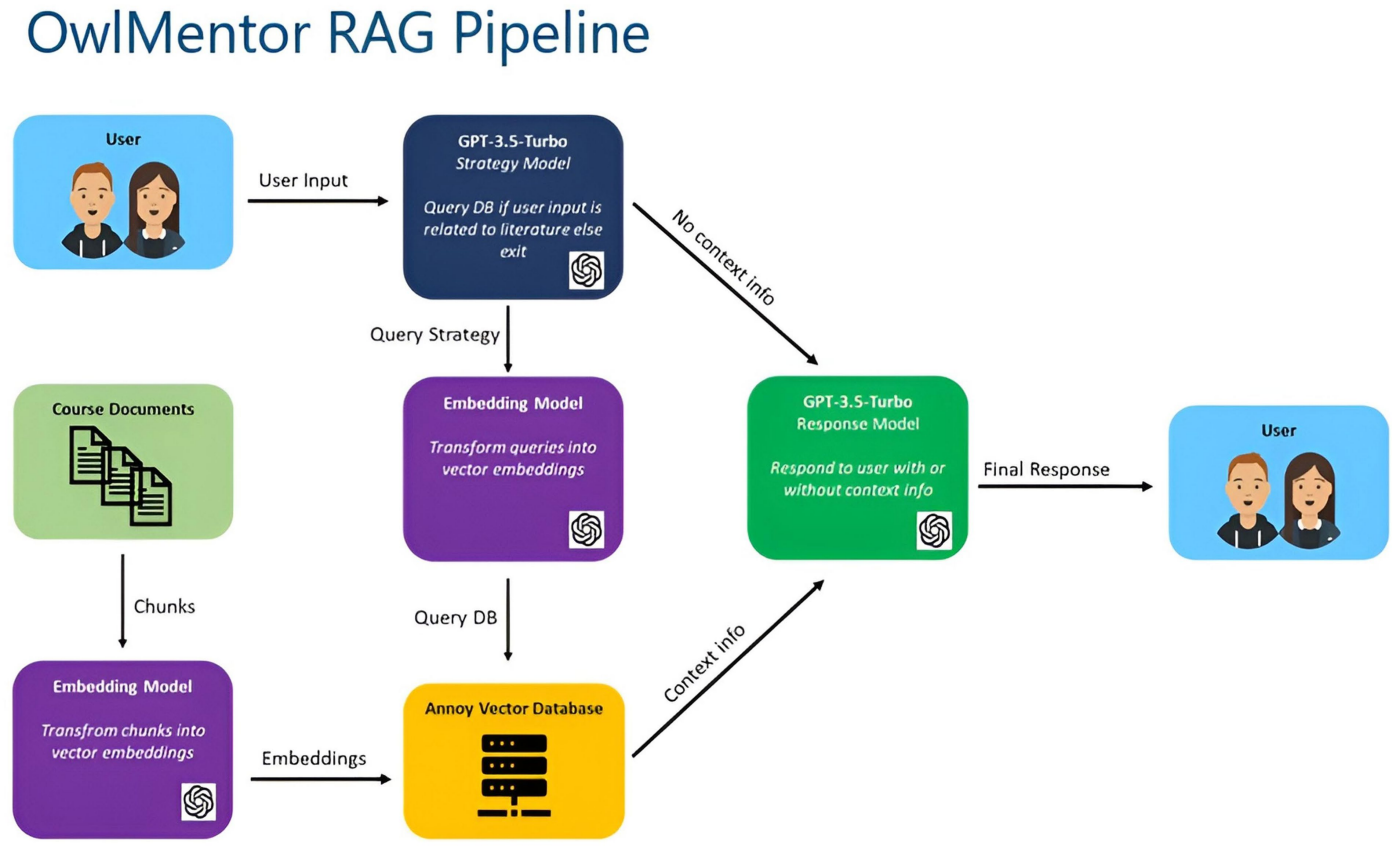
Application dialog RAG pipeline.

To generate these vector representations, OwlMentor utilizes OpenAI’s text-embedding-ada-002 model. Documents are segmented into chunks of approximately 300 words with 20-word overlaps to maintain context across sections. The Natural Language Toolkit (NLTK) ensures that sentences are not split during this process, preserving the integrity of the text. The embeddings are stored in an Annoy-based vector index with 30 trees and an embedding dimension of 1,536. This vector index serves as a critical component of the RAG system (Figure 7), enabling fast and accurate retrieval of relevant document sections in response to user queries. The RAG system is central to OwlMentor’s ability to provide context-specific assistance. When a user submits a query, the system first determines whether it pertains to a relevant document within the vector database. If so, the RAG system applies logical routed retrieval by navigating the document’s structure and metadata (e.g., section titles and key topics) to identify the most pertinent sections. This focused retrieval enhances accuracy by narrowing the search to the most relevant parts of the document. The Strategy Model, a LLM (GPT 3.5-Turbo 0613) with specific instructions, further refines the retrieval process. It assesses whether the user’s query is relevant to the document’s key topics. If so, it uses logical routed retrieval to direct the query to the most relevant sections. For non-relevant queries, the Strategy Model produces direct outputs like “[EXIT]” or “[INFO],” indicating no retrieval is needed. For relevant queries, it generates refined search queries through query decomposition, breaking down complex queries into simpler, more focused ones, ensuring the most relevant sections are returned for further processing. The retrieved document sections are then passed to the Response Model, which generates responses that are aligned with the user’s query and the content of the document. This approach reduces the risk of hallucinations or irrelevant information, ensuring that the system consistently produces responses grounded in the provided course material. The temperature setting of the LLM is configured to 0.1 for the Response Model, and to 0 for the Strategy Model and for feedback generation. This ensures that responses remain precise and grounded in the provided information. The maximum tokens parameter is set to 800 for Response Model, 200 for the Strategy Model, and 250 for feedback. The streaming option is enabled to enhance response times, except for the Strategy Model.

OwlMentor’s frontend architecture is developed using React JS, ensuring a responsive and interactive user experience. This frontend enables seamless navigation between different functionalities, dynamic content rendering, and real-time updates to track user activity. Detailed lists of backend and frontend dependencies are provided in Appendices 4, 5, offering comprehensive technical insights into OwlMentor’s development.

### 2.6 Instruments

#### 2.6.1 Pre-and posttest

The pre-and posttest consist of the same 10 items: two items on each of the following five topics of the Multimedia Learning II course: Expertise-Reversal Principle, Split-Attention Principle, Worked Example Principle, Principles based on Social Cues and Emotional Design Principle (Mayer and Fiorella, 2021). For each topic, two questions were carefully crafted based on specific levels of the education objectives taxonomy (Bloom, 1956). One question was designed to assess the basic level of ‘remembering’, while the other was designed to assess the higher levels, specifically ‘understanding’ or ‘applying’. With regard to the question format, the pre-posttest consists of four multiple choice (MC) and six open questions (OP). Example items include: “What is the split-attention effect? Select the correct answer (Remembering, MC),” “In a study of science learning by Leslie et al. (2012), more experienced students learned better from listening-only texts, while novices benefited more from audiovisual presentations. How would you explain these results using the principle of reversal of subject knowledge? (Understanding, OP)” or “‘As a primary school teacher who wants to teach the concept of addition using worked examples, how could you improve the effectiveness of your worked examples? (Applying, OP). The pretest was carried out at the beginning of the seminar and the posttest one week before the final exam.

#### 2.6.2 Questionnaire TAM (perceive ease of use, perceived usefulness, intention to use) + self-efficacy

A structured self-assessment questionnaire (for complete questionnaire see Appendix) was used to evaluate the constructs of the technology acceptance model (Perceived Ease of Use, Perceived Usefulness, Intention to Use) and Self-Efficacy. The questionnaire contained proven scales that were selected and modified from the existing literature. Perceived Ease of Use was assessed using a four-item scale derived from Venkatesh and Davis (2000), which showed Cronbach’s alpha values between *α* = 0.86 and α = 0.98, as stated by Venkatesh and Davis (2000). Sample items include “I find OwlMentor easy to use” and “Interacting with OwlMentor does not require much attention.” The scale for Perceived Usefulness was adapted from a three-item scale by Liaw (2008), which had a Cronbach’s alpha reliability of α = 0.90. A sample item for Perceived Usefulness is “I believe OwlMentor is a useful learning tool.” Intention to Use was measured by using a three-item scale from Liaw (2008) with a Cronbach’s alpha of approximately α = 0.89. Sample items are “I intend to use OwlMentor’s content to assist my learning” or “I intend to use OwlMentor to assist my learning in the future.” The responses on all scales were recorded on a seven-point Likert scale ranging from “strongly disagree” to “strongly agree.” Moreover, Self-Efficacy was measured via self-reports. For this purpose, we used the nine-item scale by Pintrich and De Groot (1990), which has an internal consistency of Cronbach’s alpha = 0.89 to 0.92 (Pintrich and De Groot, 1990). Example items are “I am certain I can understand the ideas taught in this course.” or “I know that I will be able to learn the material for this class.” For the Self-Efficacy scale, we used the original 5-point Likert-scale ranging from “strongly disagree” to “strongly agree.”

#### 2.6.3 Reliability

The reliability of the measurement instruments was assessed using Cronbach’s alpha (α). The reliability for Perceived Usefulness ranged from *α* = 0.92 to *α* = 0.97 across the three time points. Reliability for Perceived Ease of Use ranged from *α* = 0.84 to *α* = 0.96. Intention to Use showed a reliability range from *α* = 0.92 to *α* = 0.99. Self-Efficacy showed a high reliability with a range of *α* = 0.93 to *α* = 0.98. For the pretest, the reliability was α = 0.78, for the posttest α = 0.80. These values show that the measurement instruments used in the study were consistently reliable across the various constructs and time points.

### 2.7 Log data/ system use

During use, we saved the conversations, dialogs, and automatically generated questions for qualitative analyses. We also collected the following quantitative log data to quantify the Actual System Use: Number of conversations created, number of dialogs conducted, number of questions generated, number of questions deleted, number of quizzes created, number of quizzes completed, number of feedback received. Each of these quantitative log data represents a user activity. We calculate the Actual System Use by adding up all these user actions to one value. Furthermore, the use of OwlMentor was anonymous and no personal data was collected.

### 2.8 Procedure

**Figure 8:**
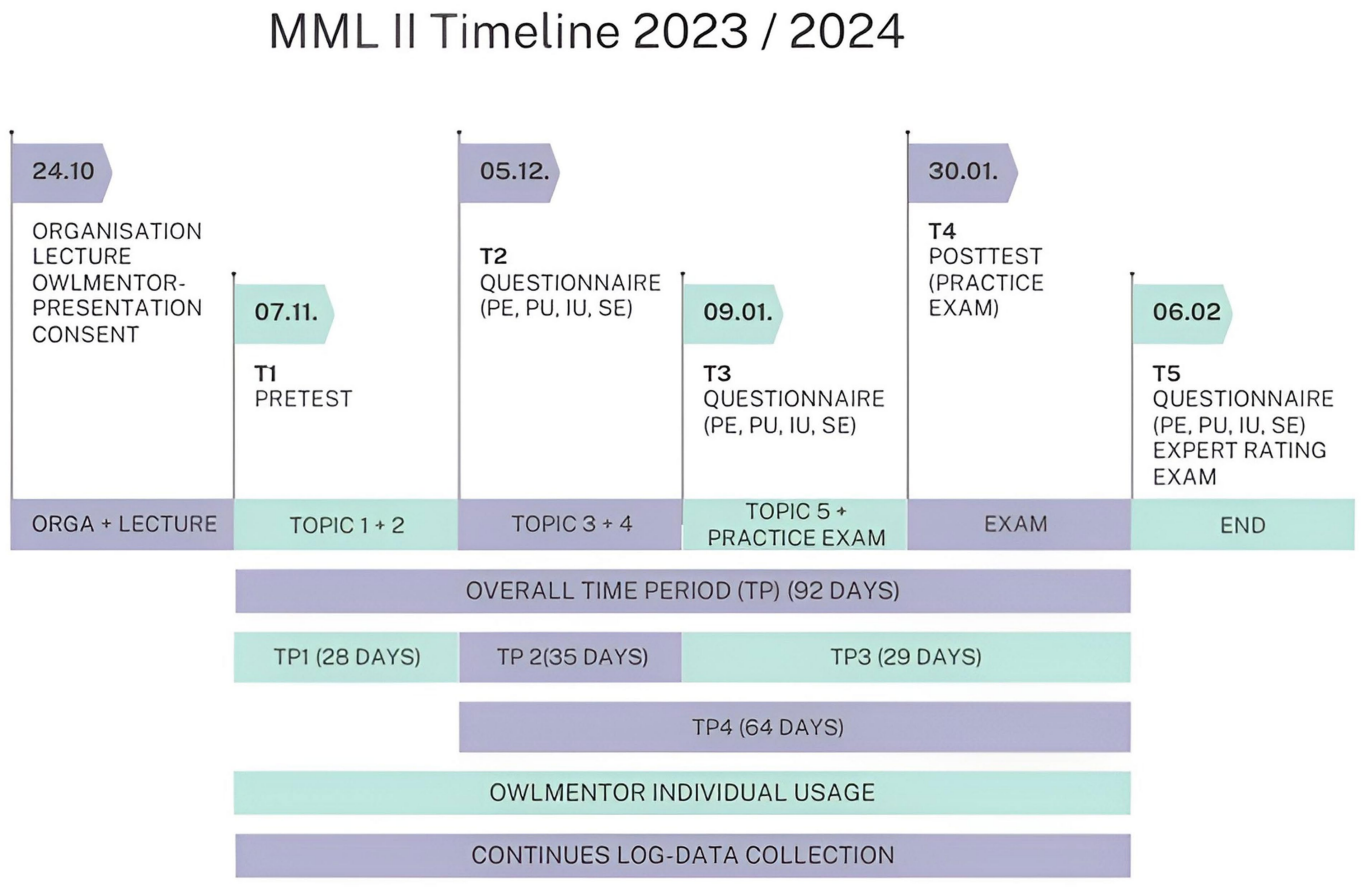
Timeline MML II.

First, the revised version of the OwlMentor was presented to the students as part of the Multimedia Learning II seminar and they were asked to use the OwlMentor throughout the course. Figure 8 shows a timeline of the course details and the measurement times. The use of the OwlMentor was voluntary and anonymous. The participants first signed a declaration of consent for the study. They then completed the pretest under the supervision of the course instructors. The pre-test and post-test were designed to assess students’ understanding of the key principles covered in the Multimedia Learning II course. The instructors first created a series of questions based on the course literature relevant to the exam and covering the different levels of Bloom’s Taxonomy (Bloom, 1956). After a collaborative review, the lecturers selected two questions per topic, which were then formatted into a digital questionnaire using MS Forms. This structured assessment allowed for a sample-based evaluation of students’ baseline knowledge and learning gains in key topic areas. Following the pretest, the students were then granted anonymous access to OwlMentor. To facilitate use and ensure effective interaction with the OwlMentor, a short user manual and instructional videos were made available on the digital course platform. The evaluation of the acceptance of the platform was carried out using the TAM questionnaire, which was completed at three points during the course: after completion of the second topic (T2), after the fourth topic (T3) and after the final exam at the end of the seminar (T5). The posttest was carried out at T4 under the supervision of the course instructors as a practice exam.

### 2.9 Analysis

We conducted a comprehensive analysis that included descriptive analysis, hypothesis-testing, dialog functionality evaluation, and explorative analysis. Descriptive analyses were conducted to summarize data on platform usage, questionnaire responses, and pre-and post-test performance.

Hypothesis testing included correlation analyses and linear regression. For H1 (Perceived Ease of Use and Intention to Use) and H2 (Perceived Usefulness and Intention to Use), correlation analyses were performed at three time points (T2, T3, T5). To test if Perceived Usefulness and Perceived Ease of Use are significant predictors of the Intention to Use, a multiple regression analysis was conducted. The dependent variable was Intention to Use, while the independent variables were Perceived Usefulness, Perceived Ease of Use, and time. For H3 (Intention to Use and Actual System Use), We conducted a series of linear regression analyses to investigate the relationship between Intention to Use and subsequent periods of Actual System Use. We measured Actual System Use during three time periods (TP2, TP3, TP4) and Intention to Use at two points in time (T2, T3). Specifically, we examined how Intention to Use at T2 predicted Actual System Use at TP2 and TP3, and how Intention to Use at T3 predicted Actual System Use at TP4.

- Regression 1: Intention to Use at T2 predicting Actual System Use during TP2
- Regression 2: Intention to Use at T2 predicting Actual System Use during TP3
- Regression 3: Intention to Use at T2 predicting Actual System Use during TP4
- Regression 4: Intention to Use at T3 predicting Actual System Use during TP3

These analyses were conducted to determine whether Intention to Use significantly predicted subsequent Actual System Use. H4 (Actual System Use and learning gain) was tested using a linear regression for Actual System Use in period 1 with the dependent variable learning gain and the independent variable Actual System Use.

The dialog functionality analysis consisted of two parts: categorizing user requests and conducting an expert evaluation of OwlMentor’s responses. When evaluating dialog functionality, user requests were categorized to understand the nature of interactions on the platform. The user requests were categorized into seven main categories, along with an “Other” category for requests that did not fit into the predefined categories. The categories are as follows in Table 1:

**Table 1:** User request categories for dialog functionality analysis.

Category	Description
Concept explanations and definitions	This category includes requests seeking clear definitions, descriptions, or explanations of specific terms, principles, or concepts. Users wanted basic or detailed information about certain topics to improve their understanding.
Summarization requests	This category includes requests that seek brief summaries, key points, or essential takeaways from a text, chapter, or study. Users wanted a condensed version of the content, often in the form of sentences or bullet points, to get a quick overview.
In-depth explanations	This category includes requests that seek comprehensive explanations or detailed information about specific concepts, principles, or research findings. Users wanted to gain a deeper understanding of complex topics, often aiming to go beyond surface-level definitions.
Relationship and connection	This category includes requests that seek to understand the relationships or connections between different concepts, principles, or effects. Users wanted to comprehend how various elements interact with each other and what impacts they have on one another.
Importance and rationale	This category includes requests that seek the reasons or justification for the importance of a study, paper, or specific information. Users wanted to understand why something is important and what impact or relevance it holds.
Targeted information requests	This category includes requests that seek specific information, concepts, or variables. Users wanted to obtain targeted data or key concepts about a topic, often in the form of lists or brief descriptions.
Practical examples and applications	This category includes requests that seek practical examples or applications of specific concepts, methods, or principles. Users wanted to see concrete examples to better understand abstract ideas or to know how to apply them in practice.
Other	This category includes requests that did not fit into the specific previously defined categories.

Furthermore, two experts reviewed all dialogs between users and OwlMentor and evaluated OwlMentor’s responses. Therefore, the experts rated the responses using a scale where 1 indicates “very good” and 6 indicates “unsatisfactory.” The inter-rater reliability was measured using ICC3 indicating good reliability between the raters with a value of 0.82.

Finally, we examined the influence of Self-Efficacy on other variables measured in the study using correlational analysis. This included analyzing the influence of Self-Efficacy on Perceived Ease of Use, Perceived Usefulness, and Intention to Use at three measurement time points (T2, T3, T5), as well as examining the relationship between Self-Efficacy and Actual System Use throughout the course duration.

## 3 Results

In this section, we present the results of the study, focusing on the reliability of the measurement instruments, descriptive analysis of OwlMentor usage and questionnaire responses, pre-and post-test performance, hypothesis testing, and an exploratory analysis of the dialog functionality and the influence of self-efficacy.

### 3.1 Descriptive analysis

In this subsection, we provide a detailed analysis of the descriptive statistics for OwlMentor usage, responses from the questionnaire, and pre-and post-test performance. This includes an overview of the engagement patterns and self-reported measures from participants throughout the study period.

#### 3.1.1 OwlMentor usage

Table 2 presents the usage statistics of the OwlMentor application over four different periods (Overall, TP1, TP2, TP3). Overall usage across the entire course duration shows that the most frequently used functions were messages indicating that students primarily engaged with the platform through this interaction. As can be seen from the table, engagement was highest in TP1, while it decreased significantly in TP2. However, there was an increase in engagement from TP2 to TP3, which was closer to the final exam. This pattern can be observed across different measures such as conversations, messages, and questions created. User activity was most frequent in TP1, but despite the initial decrease in TP2, user activity increased again in TP3. In addition, user engagement was higher in the first period with 11 active users compared to only 6 active users in each of the following periods.

**Table 2:** OwlMentor usage statistics.

*N* = 16	Time period	Overall	TP1	TP2	TP3
Actual usage	*M*	21.38	9.38	4.44	7.56
	*SD*	32.64	10.82	9.20	15.21
	Sum	342	150	71	121
Conversations	*M*	2.25	1.00	0.56	0.69
	*SD*	2.84	1.10	1.03	1.25
	Sum	36	16	9	11
Messages	*M*	15.13	6.25	3.00	5.88
	*SD*	24.21	7.11	6.15	13.79
	Sum	242	100	48	94
Message likes	*M*	0.38	0.25	0.06	0.06
	*SD*	1.03	1.00	0.25	0.25
	Sum	6	4	1	1
Questions	*M*	1.88	1.00	0.5	0.38
	*SD*	3.07	2.03	1.41	0.89
	Sum	30	16	8	6
Temporary questions	*M*	0.56	0.19	0.13	0.25
	*SD*	1.55	0.75	0.50	0.58
	Sum	9	3	2	4
Deleted questions	*M*	0.31	0.25	0	0.06
	*SD*	0.70	0.58	0	0.25
	Sum	5	4	0	1
Practiced questions	*M*	0.88	0.44	0.19	0.25
	*SD*	2.63	1.75	0.75	1.00
	Sum	14	7	3	4

No quizzes were created or completed and no feedback was received.

#### 3.1.2 Questionnaire

The questionnaire was used to measure Perceived Ease of Use, Perceived Usefulness, Intention to Use, and Self-Efficacy at three time points (T2, T3, T5). As shown in the questionnaire data in Table 3, mean Perceived Usefulness decreased from T2 to T3 and continued to decrease from T3 to T5, moving from somewhat disagree and neutral at T2 and T3 to somewhat disagree and disagree at T5. Perceived Ease of Use increased from T2 to T3, but then decreased from T3 to T5. Intention to Use remained stable from T2 to T3 but decreased from T3 to T5. Self-Efficacy remained relatively stable across all three time points and showed only minimal changes. For the users of the AI-based learning platform, the mean Perceived Usefulness initially increased from T2 to T3 and then decreased from T3 to T5. Perceived Ease of Use showed an increase from T2 to T3 and a slight decrease from T3 to T5. Intention to Use remained relatively stable from T2 to T3, with a slight decrease from T3 to T5. Self-Efficacy remained relatively stable across all three time points with minimal changes. For the non-users, mean Perceived Usefulness decreased consistently from T2 to T5. Perceived Ease of Use increased from T2 to T3 and then decreased at T5. Intention to Use showed a decreasing trend from T2 to T5. Self-Efficacy remained relatively stable, with a decrease from T2 to T3 and slight increase from T3 to T5. Comparing users and non-users, users generally reported higher Perceived Usefulness and Intention to Use at all three time points, while non-users showed a more pronounced decrease in Perceived Usefulness over time. Perceived Ease of Use increased similarly for both groups from T2 to T3, but users maintained higher Perceived Ease of Use at T5 compared to non-users. Self-Efficacy was slightly higher for non-users at all time points, although the differences were minimal.

**Table 3:** Questionnaire data.

Group	Variable	T2 (*n* = 15) *M (SD)*	T3 (n = 13) *M (SD)*	T5 (n = 10) *M (SD)*
All users	PU	3.66 (1.64)	3.36 (1.79)	2.73 (1.34)
	PE	4.27 (1.73)	5.04 (1.07)	4.25 (1.49)
	IU	2.82 (1.86)	2.72 (1.77)	2.10 (1.56)
	SE	3.66 (1.19)	3.58 (0.78)	3.57 (0.80)
Users	PU	3.80 (1.41) (*n* = 10)	3.85 (1.88) (*n* = 9)	3.19 (1.36) (*n* = 7)
	PE	4.05 (1.73) (*n* = 10)	4.97 (1.23) (*n* = 9)	4.46 (1.70) (*n* = 7)
	IU	2.87 (1.55) (*n* = 10)	3.00 (1.95) (*n* = 9)	2.33 (1.76) (*n* = 7)
	SE	3.40 (1.29) (*n* = 10)	3.57 (0.88) (*n* = 9)	3.43 (0.92) (*n* = 7)
None users	PU	3.40 (2.20) (*n* = 5)	2.25 (1.00) (*n* = 4)	1.67 (0.33) (*n* = 3)
	PE	4.70 (1.86) (*n* = 5)	5.19 (0.66) (*n* = 4)	3.75 (0.87) (*n* = 3)
	IU	2.73 (2.58) (*n* = 5)	2.08 (1.26) (*n* = 4)	1.56 (0.96) (*n* = 3)
	SE	4.18 (0.85) (*n* = 5)	3.61 (0.58) (*n* = 4)	3.89 (0.30) (*n* = 3)

Perceived usefulness (PU), perceived ease of use (PE), intention to use (IU): 1 = strongly disagree, 7 = strongly agree, self-efficacy (SE): 1 = strongly disagree, 5 = strongly agree.

#### 3.1.3 Pre- posttest performance

The results before and after the test show that users’ overall performance improved significantly after taking part in the course, *t*(13) = −3.56, *p* < 0.001, *Cohen’s d* = 1.93. There were improvements in all areas of the individual topics, with users scoring higher in the post-test than in the pre-test. The increases were more pronounced for some topics than for others, which is mainly due to lower values in the pretest. The detailed statistics in the Table 4 provide a comprehensive overview of these improvements and illustrate the participants’ overall learning progress in the different subject areas during the course.

**Table 4:** Pre- posttest performance all users.

All users *M (SD)*	Pre (*n* = 14)	Post (*n* = 14)	Diff (*n* = 14)
Overall	4.68 (3.07)	11.57 (3.62)	6.89 (3.57)
Topic 1	1.04 (0.41)	2.14 (0.74)	1.11 (0.81)
Topic 2	1.14 (1.03)	2.21 (1.03)	1.07 (1.40)
Topic 3	1.29 (1.07)	2.18 (0.91)	0.89 (1.30)
Topic 4	0.68 (1.01)	2.71 (1.01)	2.04 (1.03)
Topic 5	0.54 (0.50)	2.32 (0.99)	1.79 (1.07)

Table 5 shows the results of the pre-and post-tests as well as the difference for users. For users of the platform, overall performance improved from the pre-test to the post-test, with substantial gains across all topics. The learning gains for users were consistent across the different subject areas.

**Table 5:** Pre- Posttest Performance users.

Users *M (SD)*	Pre (*n* = 10)	Post (*n* = 10)	Diff (*n* = 10)
Overall	4.95 (3.46)	12.30 (3.34)	7.35 (3.85)
Topic 1	1.00 (0.47)	2.25 (0.79)	1.25 (0.86)
Topic 2	1.30 (1.06)	2.45 (0.90)	1.15 (1.56)
Topic 3	1.20 (1.23)	2.30 (0.95)	1.10 (1.45)
Topic 4	0.90 (1.13)	3.00 (0.58)	2.10 (0.94)
Topic 5	0.55 (0.50)	2.30 (1.01)	1.75 (1.03)

Non-users also exhibited performance improvements from the pre-test to the post-test, though the overall gains were slightly lower compared to users (Table 6). Non-users showed improvements on all topics with varying degrees of progress in each topic. Comparing users and non-users, users generally demonstrated higher overall learning gains across all topics. Both groups showed improvement, but users had more pronounced gains in most topics. Non-users also improved, but their performance increases were generally lower than those of the users.

**Table 6:** Pre- posttest performance none users.

None users *M (SD)*	Pre (*n* = 4)	Post (*n* = 4)	Diff (*n* = 4)
Overall	4.00 (2.00)	9.75 (4.13)	5.75 (2.90)
Topic 1	1.13 (0.25)	1.88 (0.63)	0.75 (0.65)
Topic 2	0.75 (0.96)	1.63 (1.25)	0.88 (1.03)
Topic 3	1.50 (0.58)	1.88 (0.85)	0.38 (0.75)
Topic 4	0.13 (0.25)	2.00 (1.58)	1.88 (1.38)
Topic 5	0.50 (0.58)	2.38 (1.11)	1.88 (1.32)

### 3.2 Hypothesis testing

In this section, we present the results of our hypothesis testing. We examined four hypotheses related to the relationships between Perceived Ease of Use, Perceived Usefulness, Intention to Use, Actual System Use, and learning gains. The findings for each hypothesis are detailed below.

#### 3.2.1 H1 and H2

H1 stated that Perceived Ease of Use is positively related to the Intention to Use. Perceived Ease of Use and Intention to Use were measured at three time points, and the correlations between them were analyzed. At T2, there was a significant positive correlation between Perceived Ease of Use and Intention to Use [*r*(15) = 0.66, *p* = 0.007], indicating that higher Perceived Ease of Use was associated with a higher Intention to Use. This correlation suggests a strong, positive relationship between the two variables at this time point. However, at T3, no significant correlation was found [*r*(13) = 0.01, *p* = 0.984], indicating no relationship between Perceived Ease of Use and Intention to Use at this time point. Similarly, at T5, there was no significant correlation [*r*(10) = 0.22, *p* = 0.533], suggesting that Perceived Ease of Use did not significantly relate to Intention to Use at this later time point. Based on these results, the hypothesis is partly confirmed as a significant positive association between Perceived Ease of Use and Intention to Use was only observed at T2.

H2 stated that Perceived Usefulness is positively related to Intention to Use. Significant positive correlations between Perceived Usefulness and Intention to Use were found for all three time points. At T2, there was a significant positive correlation between Perceived Usefulness and Intention to Use [*r*(15) = 0.94, *p* < 0.001], indicating that higher Perceived Usefulness was strongly associated with a higher Intention to Use. At T3, a significant positive correlation was found [*r*(13) = 0.79, *p* < 0.001], indicating a strong relationship between Perceived Usefulness and Intention to Use at this time point. Similarly, at T5, there was a significant positive correlation [*r*(10) = 0.87, *p* < 0.001], suggesting that higher Perceived Usefulness continued to be strongly associated with higher Intention to Use. Based on these results, the hypothesis is confirmed.

A multiple regression analysis was conducted to examine whether Perceived Usefulness and Perceived Ease of Use significantly predict the Intention to Use (Intention to Use), controlling for time (Time). The overall model was significant, *F* (3, 34) = 37.541, *p* < 0.001, and accounted for approximately 76.8% of the variance in Intention to Use, with *R2* = 0.768. The regression coefficients indicated that Perceived Usefulness was a significant predictor of Intention to Use (*β* = 0.950, *t* = 9.336, *p* < 0.001), suggesting that higher Perceived Usefulness is associated with higher Intention to Use. However, Perceived Ease of Use (*β* = −0.014, *t* = −0.133, *p* = 0.895) and Time (*β* = 0.090, *t* = 0.493, *p* = 0.625) were not significant predictors of Intention to Use.

#### 3.2.2 H3

H3 stated that higher Intention to Use leads to higher Actual Usage. A series of linear regression analyses were conducted to examine the predictive relationship between Intention to Use and subsequent Actual System Use periods. For the first regression, Intention to Use at T2 did not significantly predict Actual System Use during TP2, *F* (1, 13) = 0.487, *p* = 0.497. The model explained only 3.6% of the variance (*R*2 = 0.036, adjusted *R2* = −0.038). The second regression analysis indicated that Intention to Use at T2 did not significantly predict Actual System Use during TP3, *F* (1, 13) = 1.477, *p* = 0.246. This model explained 10.2% of the variance (*R2* = 0.102, adjusted *R2* = 0.033). In the third regression, Intention to Use at T2 was not a significant predictor of Actual System Use during TP4, *F* (1, 13) = 1.136, *p* = 0.306. The model explained 8% of the variance (*R2* = 0.080, adjusted *R2* = 0.010). However, the fourth regression analysis revealed that Intention to Use at T3 significantly predicted Actual System Use during TP3, *F* (1, 13) = 10.730, *p* = 0.007. This model accounted for 49.4% of the variance (*R2* = 0.494, adjusted *R2* = 0.448). Based on these results, Hypothesis 3 is partially confirmed. While Intention to Use at T3 significantly predicted Actual System Use during TP3, Intention to Use at T2 did not significantly predict Actual System Use at TP2, TP3, or TP4.

#### 3.2.3 H4

H4 stated that higher usage of the OwlMentor leads to higher learning gains. The difference between pre-and post-test scores was calculated as the learning gain, and the Actual System Use for the complete period of the course was analyzed. A simple linear regression analysis was conducted to examine whether the Actual System Use significantly predicts the learning gain for the users of the application. The results of the regression indicated that Actual System Use was not a significant predictor of learning gain, *F* (1, 8) = 0.330, *p* = 0.581, and explained only 4.0% of the variance (*R2* = 0.040, adjusted *R2* = −0.080). Based on these results, the hypothesis is not confirmed.

### 3.3 Dialog functionality analysis

106 user requests were categorized into seven main categories, along with an “Other” category for requests that did not fit into the predefined categories. The frequencies of these categories are as shown in Table 7. Furthermore, an expert evaluation was conducted to assess the quality of OwlMentor’s responses. The mean rating given to 106 OwlMentor responses was 1.59 with a standard deviation of 0.94, suggesting that the overall quality of the responses was rated between “very good” and “good.”

**Table 7:** Distribution of user requests.

Category	Number of requests
Concept explanations and definitions	40
Summarization requests	23
In-depth explanations	14
Relationship and connection	8
Importance and rationale	7
Targeted information requests	6
Practical examples and applications	6
Other	2

### 3.4 Explorative analysis

Due to the overall low usage of our application, the negative correlation between Perceived Usefulness and Intention to Use and the low Perceived Usefulness values combined with stable and high Self-Efficacy values over time, an exploratory analysis was conducted. The explorative assumption was that Self-Efficacy has a significant influence on how useful the application is perceived, as well as on its intended and Actual System Use. In this exploratory analysis, we investigated the influence of Self-Efficacy on Perceived Usefulness, Perceived Ease of Use, Intention to Use, and Actual System Use.

#### 3.4.1 Self-efficacy and perceived usefulness

At T2, the correlation between Self-Efficacy and Perceived Usefulness was positive but not significant [*r*(15) = 0.33, *p* = 0.235], indicating that higher Self-Efficacy was weakly positive associated with Perceived Usefulness at this time point. At T3, there was a negative correlation between Self-Efficacy and Perceived Usefulness [*r*(13) = −0.53, *p* = 0.061], suggesting a moderate association of higher Self-Efficacy and lower Perceived Usefulness, although this relationship was not statistically significant. By T5, the negative correlation between Self-Efficacy and Perceived Usefulness was significant [*r*(10) = −0.85, *p* = 0.002], indicating that higher Self-Efficacy was strongly associated with lower Perceived Usefulness at this later time point.

#### 3.4.2 Self-efficacy and perceived ease of use

At T2, there was a significant positive correlation between Self-Efficacy and Perceived Ease of Use [*r*(15) = 0.61, *p* = 0.016], indicating that higher Self-Efficacy was associated with higher Perceived Ease of Use at this time point. At T3, the correlation between Self-Efficacy and Perceived Ease of Use became negative but was not significant [*r*(13) = −0.17, *p* = 0.587], suggesting that higher Self-Efficacy was not significantly associated with lower Perceived Ease of Use at this time point. At T5, the negative correlation remained but was not significant [*r*(10) = −0.21, *p* = 0.566], indicating that higher Self-Efficacy continued to show a non-significant tendency towards lower Perceived Ease of Use.

#### 3.4.3 Self-efficacy and intention to use

At T2, the correlation between Self-Efficacy and Intention to Use was positive but not significant [*r*(15) = 0.23, *p* = 0.414], indicating that higher Self-Efficacy was not significantly associated with higher Intention to Use at this time point. At T3, a significant negative correlation was observed between Self-Efficacy and Intention to Use [*r*(13) = −0.59, *p* = 0.035], suggesting that higher Self-Efficacy was associated with lower Intention to Use at this time point. By T5, this negative correlation became even more pronounced and significant [*r*(10) = −0.81, *p* = 0.005], indicating that higher Self-Efficacy was strongly associated with lower Intention to Use at this later time point.

#### 3.4.4 Self-efficacy and actual system use

There was a negative correlation between Self-Efficacy at T2 and Actual System Use during both subsequent time periods (TP4, TP2). The correlation between Self-Efficacy at T2 and Actual System Use during TP2 was significant [*r*(15) = −0.52, *p* = 0.047], suggesting that higher Self-Efficacy was significantly associated with lower actual usage at TP2. However, the correlation between Self-Efficacy at T2 and Actual System Use for TP4 was negative but not significant [*r*(15) = −0.48, *p* = 0.071], indicating that higher Self-Efficacy was weakly associated with lower Actual System Use at TP4. There was a significant negative correlation between Self-Efficacy at T3 and Actual System Use during TP3 [*r* (13) = −0.65, *p* = 0.016], indicating that higher Self-Efficacy was significantly associated with lower actual usage during TP3.

Additionally, there was a negative correlation between Self-Efficacy at all three time points (T2, T3, T5) and the Actual System Use for the overall TP. Specifically, there was a significant negative correlation between Self-Efficacy at T2 and Actual System Use for the overall TP [*r*(15) = −0.54, *p* = 0.037], between Self-Efficacy at T3 and Actual System Use for the overall TP [*r*(13) = −0.67, *p* = 0.012], and between Self-Efficacy at T5 and Actual System Use for the overall TP [*r*(10) = −0.76, *p* = 0.010], indicating that higher Self-Efficacy at each of these time points was significantly associated with lower overall Actual System Use.

## 4 Discussion

In the following section, we provide a detailed discussion of the key findings from this study, examine its limitations, offer an outlook on potential directions for future research and development, and present our conclusions.

### 4.1 Discussion of key findings

In this discussion, we analyze the results of OwlMentor’s use and impact in a university course, where it was utilized to help students understand scientific texts. We examine the relationship between the individual variables of the TAM model across three measurement points, showing that the assumptions of the TAM model do not always hold true in every case and suggesting the need for a more flexible approach in the future. Specifically, the relationships between Perceived Ease of Use, Intention to Use, and Actual System Use are more complex as the TAM model suggests. Additionally, we consider the role of general Self-Efficacy when analyzing the TAM model. We also clarify the extent to which the use of OwlMentor is associated with learning gains. Finally, we consider user interactions with OwlMentor and highlight the quality of AI responses, underscoring the effectiveness of the RAG approach.

Based on the TAM (Davis et al., 1989; Venkatesh and Davis, 1996), our first hypothesis (H1) posited that Perceived Ease of Use would positively correlate with the Intention to Use OwlMentor. Our findings partially confirmed this: at the beginning of the course, there was a significant positive correlation, indicating that students who found the platform easy to use were more likely to intend to use it. However, this correlation was not significant later. Descriptive statistics showed that while Perceived Ease of Use initially increased, it slightly decreased over time, and Intention to Use remained stable initially but declined later. This aligns with Davis et al. (1989), who noted that the effect of Perceived Ease of Use on behavioral intention subsided over time. Researchers like Adams et al. (1992), Chau (1996), Gefen and Straub (2000), and Igbaria et al. (1996) suggest that the influence of Perceived Ease of Use on Intention to Use is stronger in the early stages but diminishes over time. Subramanian (1994) also found that Perceived Ease of Use has less impact on usage over time if the technology is inherently easy to use. Our results show that while Perceived Ease of Use initially influenced Intention to Use, this relationship diminished, suggesting ease of use alone is insufficient for sustained engagement. Factors like Perceived Usefulness and Self-Efficacy, related to students’ growing domain knowledge, seem to become more influential over time. Initially, students may have found OwlMentor easy to use but later realized it did not offer as much benefit as expected, or they could meet course demands without the tool. The strong, stable correlation between Perceived Usefulness and Intention to Use supports that perceived added value is crucial for sustained use. Additionally, the shift from a positive to a negative correlation between Self-Efficacy and both Perceived Usefulness and Intention to Use suggests that students with higher Self-Efficacy felt less need for the tool as the course progressed. This aligns with Davis (1985), who noted that Perceived Usefulness has more influence than Perceived Ease of Use on system acceptance. Chuttur (2009) also pointed out that external factors like system experience, level of education, and age may influence system usage, while Yousafzai et al. (2007a) proposed that moderators affecting Perceived Usefulness and Perceived Ease of Use include Self-Efficacy, experience, educational level, skills, and knowledge.

The second hypothesis (H2) posited that Perceived Usefulness would positively correlate with the Intention to Use. This hypothesis was fully confirmed, with significant positive correlations at all three time points, consistent with the TAM (Davis, 1989; Chuttur, 2009). This finding aligns with previous studies and meta-analyses showing that Perceived Usefulness is a stronger predictor of technology adoption than Perceived Ease of Use (Amoako-Gyampah and Salam, 2004; Sharp, 2006; Yousafzai et al., 2007b; Yu et al., 2024). Our results confirmed this strong correlation, indicating that students are more likely to use OwlMentor if they find it useful, underscoring its importance for technology adoption (Opoku and Enu-Kwesi, 2019; Zou and Huang, 2023). Descriptive statistics show a decline in average Perceived Usefulness over the study, suggesting students became less positive about the platform’s usefulness, contributing to the decline in Intention to Use, even though Perceived Ease of Use initially increased. This could be addressed in future by integrating regular knowledge assessments to adapt the support provided by the tool according to the students’ evolving needs. The relationship between Self-Efficacy and Perceived Usefulness further supports this interpretation. Initially, there was no significant correlation between Self-Efficacy and Perceived Usefulness, but over time, a significant negative correlation emerged. This delayed emergence could be attributed to the time students needed to familiarize themselves with the course content, the difficulty of the texts, and how well they could cope with these demands. Students could only validly assess the platform’s usefulness after thoroughly testing its capabilities and understanding the challenges posed by the learning tasks. Additionally, as students became more familiar with the course material and better understood the content over time, they may have found OwlMentor less necessary. This suggests that students with high confidence in their abilities found the course requirements manageable without OwlMentor. Consequently, they perceived the platform as less useful. These confident students likely believed they could succeed in the course without additional help, leading to a lower perceived necessity for the platform. Additionally, using OwlMentor required extra effort, and given their high Self-Efficacy, students might have decided that the time and effort needed to use the tool were not justified by its perceived benefits.

The third hypothesis (H3) proposed that Intention to Use would lead to Actual System Use. This hypothesis was only partially confirmed. During the initial phase of the study, the analysis showed that students’ Intention to Use OwlMentor did not significantly predict their Actual System Use. However, in the subsequent phase, as students became more familiar with OwlMentor, a significant positive relationship emerged. This indicates that the expected relationship between Intention to Use and Actual System Use became more evident over time, as students gained more experience with the platform. According to TAM, Perceived Ease of Use and Perceived Usefulness are important determinants of Intention to Use and Actual System Use of technologies (Davis, 1989; Venkatesh and Davis, 2000). However, our results suggest that the initial interest triggered by Perceived Usefulness did not immediately translate into Actual System Use, possibly due to students’ exploratory approach or their existing confidence in mastering the course requirements without additional tools. The negative relation of Self-Efficacy on Actual System Use also provides important context. A negative correlation between Self-Efficacy and Actual System Use was observed from the early stages, which strengthened over time. This reflects the relation of Self-Efficacy on Intention to Use, where higher Self-Efficacy was associated with lower Intention to Use and subsequently lower Actual System Use. Students with high Self-Efficacy, who were confident that they could manage the demands of the course independently, saw less need for the OwlMentor and therefore used it less frequently. To encourage use by students with high Self-Efficacy, the tool could offer low-effort, high-benefit features such as automatic checking of progress in understanding science texts. Overall, it can be said that Intention to Use did not have a strong impact on Actual System Use initially, but its influence increased as students recognized the relevance of the platform. However, students with high Self-Efficacy consistently used the platform less, probably because they felt able to succeed without its help.

The fourth hypothesis (H4) stated that higher use of the OwlMentor would lead to higher learning gains as this platform provides different functions designed to support scientific text comprehension. However, our analysis revealed no significant correlation between Actual System Use and learning gains, suggesting that the frequency of platform use alone is not sufficient to achieve higher learning gains. Another possible explanation is that students with high Self-Efficacy performed better in the post-test, which might have obscured the correlation. Future research should examine the relationship between Self-Efficacy and post-test performance more closely. This could provide further insights into how Self-Efficacy influences learning outcomes. The descriptive statistics show that both users and non-users of the OwlMentor platform experienced significant learning gains between pre-and posttest, but these gains were not directly related to the extent of platform use. This indicates that the seminar’s quality of instruction and peer presentations likely contributed primarily to the students’ learning gains. The decreasing use of OwlMentor over the course suggests that students may have relied more on other learning strategies and resources to prepare for the exam. This aligns with previous findings that high Self-Efficacy led to lower Perceived Usefulness and Intention to Use the platform. Students with high confidence in their abilities may have felt able to succeed without additional support from the AI tool, perceiving it as offering no added value, which further discouraged its use. In summary, while the course facilitated learning, OwlMentor showed promise as users demonstrated higher learning gains than non-users. Although no direct correlation between Actual System Use and learning gains was found, the descriptive results suggest the need for further experimental studies to confirm the effectiveness of AI-based tools in education. This underscores the importance of integrating AI tools into existing learning strategies and ensuring they offer clear, tangible benefits to students. The results emphasize that matching technological tools to students’ needs is crucial for their usefulness.

The analysis of OwlMentor’s dialog features offers insights into student interactions and response quality. Categorizing user requests showed a need for explanations, definitions, summaries, detailed explanations, and understanding relationships between concepts. These categories are consistent with strategies known to improve scientific text comprehension, such as self-questioning, summarizing key passages, and linking new information to prior knowledge (Gunn, 2008; Joseph and Ross, 2018; Kendeou and Van Den Broek, 2005, 2007; Sason et al., 2020). For instance, requests for concept explanations and definitions correspond to the need for understanding basic facts, concepts, and processes, which are essential for scientific literacy (Organisation for Economic Co-operation and Development (OECD), 2003; Goldman and Bisanz, 2002). Summarization requests reflect the strategy of consolidating understanding by condensing key information, aiding in the retention and comprehension of complex texts (Cromley, 2010). Requests for on-depth explanations and understanding relationships between concepts highlight students need to engage more deeply with content, similar to self-explanation methods that promote deeper learning and integration of text material (Chi et al., 1989; Chi et al., 1994; King, 1994). By supporting these strategies through its dialog function, OwlMentor helps students handle the challenges of understanding academic texts. This fits with the idea that generative AI, like large LLMs, can offer interactive and adaptable help (Gimpel et al., 2023; Kasneci et al., 2023). Such AI can make it easier for students to use complex learning strategies and improve their engagement and understanding of academic material. However, OwlMentor takes over these tasks completely, meaning that users only need to be generative in their prompting. Whether this approach is as beneficial as performing these tasks entirely by themselves is not proven and requires further experimental studies.

The expert evaluation of OwlMentor’s responses, which received positive ratings, shows that the RAG approach can effectively provide accurate and relevant responses generated by the AI. By providing specific contextual information from the course literature, the AI was able to generate accurate responses to student queries, supporting the idea that RAG systems can improve LLM performance in educational environments (Shen et al., 2023; Barnett et al., 2024). This suggests that RAG systems can support students in working with scientific texts by providing contextually informed answers, thereby addressing some of the challenges associated with understanding complex academic material. Our findings align with the theoretical promise of generative AI in education, highlighting the potential of RAG-enhanced LLMs to support students’ learning processes, and facilitate deeper engagement with course materials (Gimpel et al., 2023; Kasneci et al., 2023). However, despite the positive expert evaluations, many students did not find the system helpful. This suggests that providing accurate and relevant answers, similar to ensuring ease of use, is likely a necessary but not sufficient condition for the system to be perceived as helpful and actively used. Person variables, such as students’ expertise and Self-Efficacy, seem to play a significant role in this perception. High Self-Efficacy and advanced knowledge might reduce the perceived necessity for additional support from the AI tool, influencing its overall acceptance and usage. Therefore, while the technical performance of OwlMentor is crucial, its integration and Perceived Usefulness must also consider individual differences among students to enhance its effectiveness. Our findings illustrate how generative AI can be integrated into university courses and highlight the potential for improving the performance of such systems by providing contextual information. These insights emphasize the need for ongoing development and refinement of AI technologies like OwlMentor in educational settings to enhance their effectiveness and address their technical and pedagogical limitations (Alkaissi and McFarlane, 2023; Feldman et al., 2024).

### 4.2 Limitations

Our research question of how an AI-based learning platform to support scientific text comprehension, such as OwlMentor, can be integrated into university teaching and whether RAG systems are suitable for supporting students in their work with academic texts was partially answered. In principle, it should be noted that the results found here can provide initial indications for the integration of AI in university teaching but are severely limited in their generalizability due to methodological weaknesses. Although this study provides valuable insights into the usage behavior, perceptions, and learning outcomes associated with the AI-based learning platform OwlMentor, the following limitations must be considered when interpreting the results. The sample size of 16 participants is relatively small and limits the generalizability of the results. Furthermore, the lack of a control group makes it difficult to attribute the observed learning gains solely to the use of the AI-based learning platform. Moreover, the different features of OwlMentor cannot be considered in isolation from each other or from the overall course activities. Our pre-post intervention analysis shows the progress over time for the entire course, but it does not distinguish the impact of individual features of the platform. The study relied primarily on correlation analyses to examine relationships between variables that do not establish causal relationships. Moreover, data collected through self-report may contain response bias, as participants may give socially desirable answers or inaccurately recall their perceptions. In addition, OwlMentor usage fluctuated significantly throughout the study period, particularly at exam times, complicating the interpretation of results and suggesting that platform usage may be heavily influenced by immediate academic demands and schedules. OwlMentor was designed to enhance scientific text comprehension. If this goal was achieved is not clear, as there were no specific measures of scientific text comprehension in this study. Future research should include specific assessments of scientific text comprehension to better understand the platform’s impact on this crucial aspect of academic learning.

### 4.3 Future research and development

Future research should focus on controlled studies isolating the effects of OwlMentor’s individual features, like the dialog function and question generation, on student learning and engagement. Enhancing LLM response quality using the RAG approach and assessing the accuracy and pedagogical value of generated questions are critical areas. Investigating the role of Self-Efficacy in the use and perception of AI-powered platforms will shed light on how these tools can be tailored to students’ varying levels of confidence. It’s important to address the specific needs of students at various educational levels; undergraduates might need more support than graduates. Our study suggests that students with higher Self-Efficacy and knowledge may need different support, such as adaptive prompts or progress checks in text comprehension. Incorporating ongoing student feedback into the development process will keep the platform relevant and effective. Future development efforts for OwlMentor will focus on integrating advanced language models, such as GPT-4 or GPT-4o, to enhance the retrieval process. These models, with their larger context windows, will allow the system to provide more detailed contextual information, further improving response accuracy. Additionally, function calling will be leveraged to enable the LLM to interact more effectively with external tools. To support learning success, OwlMentor will be extended to offer targeted strategies such as summarizing sections, highlighting key points, and guided navigation through the text, offering students specific strategies for text comprehension. These features will allow students to quickly access the information they need without extensive interaction. Another improvement will simplify question generation, enabling students to create and refine questions directly within the chat interface and save them seamlessly. New question formats, including true/false and open-ended questions, will also be introduced. On the instructor side, future iterations will allow educators to integrate their own courses and materials into OwlMentor, further personalizing the platform to specific educational needs. Additionally, research should explore the impact of AI-based platforms on different student groups to ensure equitable benefits and identify necessary changes. Integrating various learning forms, such as peer or instructor collaboration, could enhance learning experiences by combining AI tools with traditional methods. To improve the TAM, future adaptations should consider the dynamic nature of user experience and expertise development. Incorporating regular assessments of domain knowledge and Self-Efficacy could enhance the Perceived Usefulness and sustained engagement with AI-based educational tools like OwlMentor. Understanding how these factors evolve over time can help in designing more adaptive and supportive learning environments. Finally, evaluating AI tools across academic disciplines will identify beneficial features for each area, informing targeted enhancements to address the unique challenges of different fields of study.

## 5 Conclusion

This study explored the integration and impact of OwlMentor, an AI-based learning platform, within a university course. Our findings indicate that the static nature of the TAM does not adequately account for the evolving influences of Perceived Ease of Use, Perceived Usefulness, and Self-Efficacy over time. While TAM partially explained initial adoption behaviors, the dynamic changes in these factors suggest the need for a more flexible approach that considers temporal shifts and the development of domain knowledge. The effectiveness of the RAG approach was demonstrated through positive expert evaluations of OwlMentor’s responses, which were accurate and relevant. However, student perceptions did not always align with these evaluations, indicating that technical performance alone is insufficient for sustained engagement. The integration of person-specific factors and adaptive functionalities is crucial for enhancing Perceived Usefulness and continued use. In conclusion, OwlMentor shows potential in supporting scientific text comprehension, but its success hinges on dynamic adaptation to user needs, continuous value addition, and integration with existing learning strategies. Future AI-enhanced educational tools must adopt a flexible, student-centered approach, emphasizing the importance of regular assessments and iterative development to remain relevant and effective in higher education.

## Data Availability

The raw data supporting the conclusions of this article will be made available by the authors, without undue reservation.
