# Supplementary material for: Exploring generative AI in higher education: a RAG system to enhance student engagement with scientific literature
Source: Front Psychol. 2024 Oct 11;15:1474892. doi: 10.3389/fpsyg.2024.1474892 (PMC11502405; doi:10.3389/fpsyg.2024.1474892)
Supplement: Supplementary file 1 [file Data_Sheet_1.docx]

Appendix 1: Questionnaire TAM + SE

| **Scales** | **Items** |
| --- | --- |
| *Perceived Ease of Use (4 Items)*  1 (strongly disagree) – 7 (strongly agree)  Cronbach´s alpha α = .92 – α = .97 | It is easy to operate OwlMentor and do what I want it to do. |
|  | I find that OwlMentor is easy to use. |
|  | I find that the interface of OwlMentor is clear and easy to understand. |
|  | I find that interacting with OwlMentor doesn’t demand much care or attention. |
| *Perceived Usefulness (3 Items)*  1 (strongly disagree) – 7 (strongly agree)  Cronbach´s alpha α = .84 – α = .96 | I believe OwlMentor is a useful learning tool. |
|  | I believe using OwlMentor is effective for learning |
|  | I believe OwlMentor's contents are informative. |
| *Intention to Use (3 Items)*  1 (strongly disagree) – 7 (strongly agree)  Cronbach´s alpha α = .92 – α = .99 | I intend to use OwlMentor to assist my learning in the future. |
|  | I intend to use OwlMentor's content to assist my learning. |
|  | I intend to use OwlMentor to improve my learning motivation. |
| *Self-Efficacy (9 Items)*  1 (strongly disagree) – 5 (strongly agree)  Cronbach´s alpha α = .93 – α = .98 | Compared with other students in this class, I expected to do well. |
|  | I am certain I can understand the ideas taught in this course. |
|  | I expect to do very well in this class. |
|  | Compared with others in this class, I think I am good student. |
|  | I am sure I can do an excellent job on the problems and tasks assigned for this class. |
|  | I think I will receive a good grade in this class. |
|  | My study skills are excellent compared with others in this class. |
|  | Compared with other students in this class, I think I know a great deal about the subject. |
|  | I know that I will be able to learn the materaial for this class. |

Appendix 2: Pre-/ Posttest

| **Topic** | **Items** |
| --- | --- |
| *1: Contiguity and Split attention* | What is the split-attention effect? Select the correct answer:   - A learning strategy that involves splitting information into smaller chunks. - A cognitive process that involves dividing attention between multiple sources of information. - A phenomenon where attention is focused on irrelevant information. - A technique used to present information in a visual format. |
|  | Name and explain a good alternative strategy to reducing the effects of split attention beyond the traditional integration strategy.   - Open answer |
| *2: Emotions in Multimedia Learning* | What is the main idea of emotional design in learning materials?   - Emotional design induces an emotional state conducive to learning. - Emotional design adds new elements to learning materials. - Emotional design enhances affective learning outcomes. - Emotional design increases the cognitive demands on learners. |
|  | Imagine you want to improve emotionally unappealing biology learning material for your students by using emotional design. Give two examples of what you could do to apply emotional design to enhance learning materials that would result in higher learning gains.   - Open answer |
| *3:* *Example-based Learning* | According to Renkl's theory of example-based learning, which approach is recommended for learners who have a rudimentary understanding of a topic, e.g. the laws of physics?   - Begin with intensive problem-solving to apply physics laws. - Employ superficial strategies like copying solutions for problem-solving. - Study and explain worked examples first, then move to problem-solving. - Prioritize rote memorization of physics laws over their practical application. |
|  | As a primary school teacher, you want to use worked examples to help your students understand the concept of addition. How could you improve the effectiveness of your worked examples?   - Open answer |
| *4: Expertise Reversal Effect* | How can detailed explanations affect knowledgeable (experienced) learners?   - Open answer |
|  | In a study on learning science topics from Leslie et al. (2012) more knowledgeable students learned better from auditory only presentation, while novices learned better from audio-visual presentation. How would you explain these results with the expertise-reversal principle?   - Open answer |
| *5: Social Cues in Multimedia Learning* | What is the personalization principle?   - Open answer |
|  | Given a multimedia learning module on the solar system, how you would use at least two types of social cues to foster generative processing in students?   - Open answer |

Appendix 3: Prompt Templates

| **Template** | **Description** | **Complete Prompt Template** |
| --- | --- | --- |
| 1. Strategy Model | The strategy model processes user queries, determining if they are relevant to the document. If relevant, it generates up to a set number of refined query strings based on the document’s key topics. The model uses metadata like section titles and key concepts to generate these queries. Irrelevant queries receive direct responses like "[EXIT]" or "[INFO]." This step ensures the query is routed correctly, improving retrieval accuracy. | You are assisting with literature research for a course. Your primary goal is to process user queries based on the specifics of a given document, especially its key topics. Document details: - Key-topics: {document_details} - Document identifier: {document_id} User's query: {user_query} Instructions: 1. Regarding Document Information: If the user seeks details about the current document, you must respond with the explicit output: Output example: "[INFO]" <= and nothing else 2. For Non-relevant Queries: If the user's query doesn't pertain to multimedia learning, cognitive load, or the provided key-topics, your direct response should be: Output example: "[EXIT]" <= and nothing else 3. For Pertinent Queries: When the query is related to the given key-topics or concepts like multimedia learning and cognitive load: - Pinpoint the main themes from the query. - Compare these themes with the key-topics. - Construct up to {max_queries} tailored query strings. - Present these in a JSON format. When formulating this response, it's imperative to use the provided document_id for the 'document' field and nowhere else. Each item should include 'query', 'document', and 'part' (the _id for the key topic as integer!). Example format: {output_example} Primary Points: - Always give preference to direct outputs like "[INFO]" or "[EXIT]" over creating queries, where relevant. - Ensure to stay within the {max_queries} boundary when formulating queries. |
| 2. OwlMentor Chat | OwlMentor’s chat functionality is tailored to respond based on the type and structure of the referenced documents. It generates precise, concise answers based on the course literature and is capable of handling a range of document types (e.g., reviews, experimental studies). The chat is designed to adapt and express uncertainty when appropriate. | You are OwlMentor, a university tutor for the Multimedia Learning course. Your responses should be informed by the course literature, taking into account the type and structure of the documents referenced. Guiding Principles: - Document Awareness: Recognize and acknowledge the type of document being referenced (literature review, book chapters, experimental studies). - Structure Adherence for Experimental Studies: When referencing experimental studies, be aware of the specific sections like abstract, theory, hypothesis, methods, results, and discussion. - Be Succinct: Offer concise, precise answers that avoid redundancy. - Express Uncertainty: If unsure, express this fact and engage in collaborative exploration with the student. - Optional: You can use bullet point lists and emojis to structure or shorten your response. Backend Processes: Understand that processes like similarity scores above 30 are internal and shouldn't interrupt the natural conversation flow. Chat History: {} Student Request: {} Document Type (if relevant): experimental study Supplementary Context Info: {} Your Answer: Craft a concise, direct response that considers the document type and its structure. Address the student’s inquiry uniquely, without reiterating prior content. Offer fresh, succinct insights or examples specific to the question and the document type in reference. |
| 3. OwlMentor Question Generation | This template enables the automatic generation of multiple-choice questions from specific document excerpts. The model reads the context, identifies key concepts, and creates a question with one correct answer and three distractors. The generated question is designed to challenge students while avoiding direct copying of text from the provided passage. This ensures high-quality question generation for practice. | Personality: Expert University Question Generator Task: systematically analyze the given context to create a single multiple-choice question. Follow this train of thought: 1. Read and Understand: First read the context to understand the key terms. 2. Identify Key Point: Derive the key idea from the text that is appropriate for the exam. 3. Formulate Question: Develop a question based on the key idea. 4. Correct Answer: Determine an answer that directly fits the context. 5. Distractors: Construct three distractors. These should be related to the general topic, but NOT directly derived from the context. Use your creativity and broader knowledge to distinguish them. 6. Explanation: Provide a brief rationale (less than 70 words) for the correct answer, referring to context as necessary. Context: '''{}''' Note: The user may have general knowledge of the topic but may not know intricate details. Make sure the question focuses on the main idea, but avoid copying sentences directly from context to avoid distractions. Instructions: Answer the question in JSON format, making sure that the "Answer" key directly matches one of the options in the "Options" key. Include a "question" key, an "options" key with 4 answer choices, an "answer" key indicating the correct option, and an "explanation" key with a short rationale. |
| 4. Question Random Search | This template is used for generating random queries across various sections of the document. The system selects random key topics from the document and formulates specific queries targeting these topics. This allows the model to generate queries based on different parts of the document, ensuring comprehensive coverage. | Given a document's key-topics, generate up to {max_queries} random queries targeting various sections in the document. Structure the output as a JSON-formatted list, associating each query with the current document and the relevant section's part/index. Input: {user_query} Document identifier: {document_id} Key-topics: {document_details} Task: 1. Randomly select topics from the document's key-topics. 2. Formulate specific queries (always in English) targeting these topics, limiting the number of queries to {max_queries}. 3. Compile results as a JSON list, with each item containing 'query', 'document' (use the provided document identifier), and 'part' (the section index). Note: Maintain the provided document identifier consistently in the 'document' field for all items in the output list. Do not exceed the specified maximum number of queries. The selection of topics for query formulation should be random to ensure diverse inquiries. |
| 5. OwlMentor Feedback | OwlMentor provides feedback on quiz questions using this prompt. The system receives a question object and the student’s answer, generating a brief, elaborative response that guides the student to a better understanding of the correct answer. | You are OwlMentor, a friendly and helpful university tutor chatbot specializing in educational psychology at Saarland University. You supervise the course Multimedia Learning in the Edutech masters program. Your task is to give short elaborative feedback on quiz questions to the student. You will be provided with the question object containing all relevant information on the question and the student's answer. question object: {} student answer: {} Provide your short feedback: |

Appendix 4: Backend Dependencies (Python)

| **Dependency** | **Version** | **Description** |
| --- | --- | --- |
| Python | 3.10 | Main language runtime |
| FastAPI | 0.95.2 | Web framework for building APIs |
| Motor | 3.1.2 | Asynchronous MongoDB driver |
| PyMongo | 4.3.3 | MongoDB driver for Python |
| Annoy | 1.17.2 | Approximate Nearest Neighbors for retrieval tasks |
| Pydantic | 1.10.8 | Data validation using Python types |
| OpenAI | 0.27.7 | API access for OpenAI models |
| NumPy | 1.24.3 | Numerical operations and array handling |
| NLTK | 3.8.1 | Natural Language Toolkit for text processing |
| Regex | 2023.x.x | Regular expression operations |
| PyJWT | 2.7.0 | JSON Web Token library for authentication |
| Python-Multipart | 0.0.6 | Handle multipart form-data |

Appendix 5: Frontend Dependencies (React JS)

| **Dependency** | **Version** | **Description** |
| --- | --- | --- |
| @babel/plugin-proposal-private-property-in-object | * | Babel plugin to transform private properties in objects for JavaScript. |
| @microsoft/fetch-event-source | ^2.0.1 | Utility for EventSource to handle server-sent events (SSE). |
| @testing-library/jest-dom | ^5.17.0 | Custom Jest matchers to test the state of the DOM for testing purposes. |
| @testing-library/react | ^13.4.0 | Library for testing React components by simulating user interactions. |
| @testing-library/user-event | ^13.5.0 | Library to simulate user events in testing scenarios. |
| @tippyjs/react | ^4.2.6 | React component for creating tooltips, popovers, and dropdowns. |
| @xstate/react | ^3.2.2 | React bindings for XState, a state management library based on statecharts. |
| axios | ^1.4.0 | Promise-based HTTP client for making API requests. |
| d3-scale-chromatic | ^3.0.0 | Color schemes for visualizations, part of D3.js. |
| react | ^18.2.0 | JavaScript library for building user interfaces. |
| react-device-detect | ^2.2.3 | Library to detect device types and render UI components accordingly. |
| react-dom | ^18.2.0 | Provides DOM-specific methods for React. |
| react-router-dom | ^6.14.2 | Library for handling routing in React applications. |
| react-scripts | 5.0.1 | Scripts and configuration used by Create React App. |
| react-select | ^5.7.4 | A flexible and customizable React select input control. |
| recharts | ^2.8.0 | A charting library built with React and D3.js. |
| web-vitals | ^2.1.4 | A set of utilities for measuring the performance of web applications. |
| xstate | ^4.38.2 | State machine and statechart library for managing state logic in JavaScript applications. |

Appendix 6: OwlMentor Versions User Interfaces


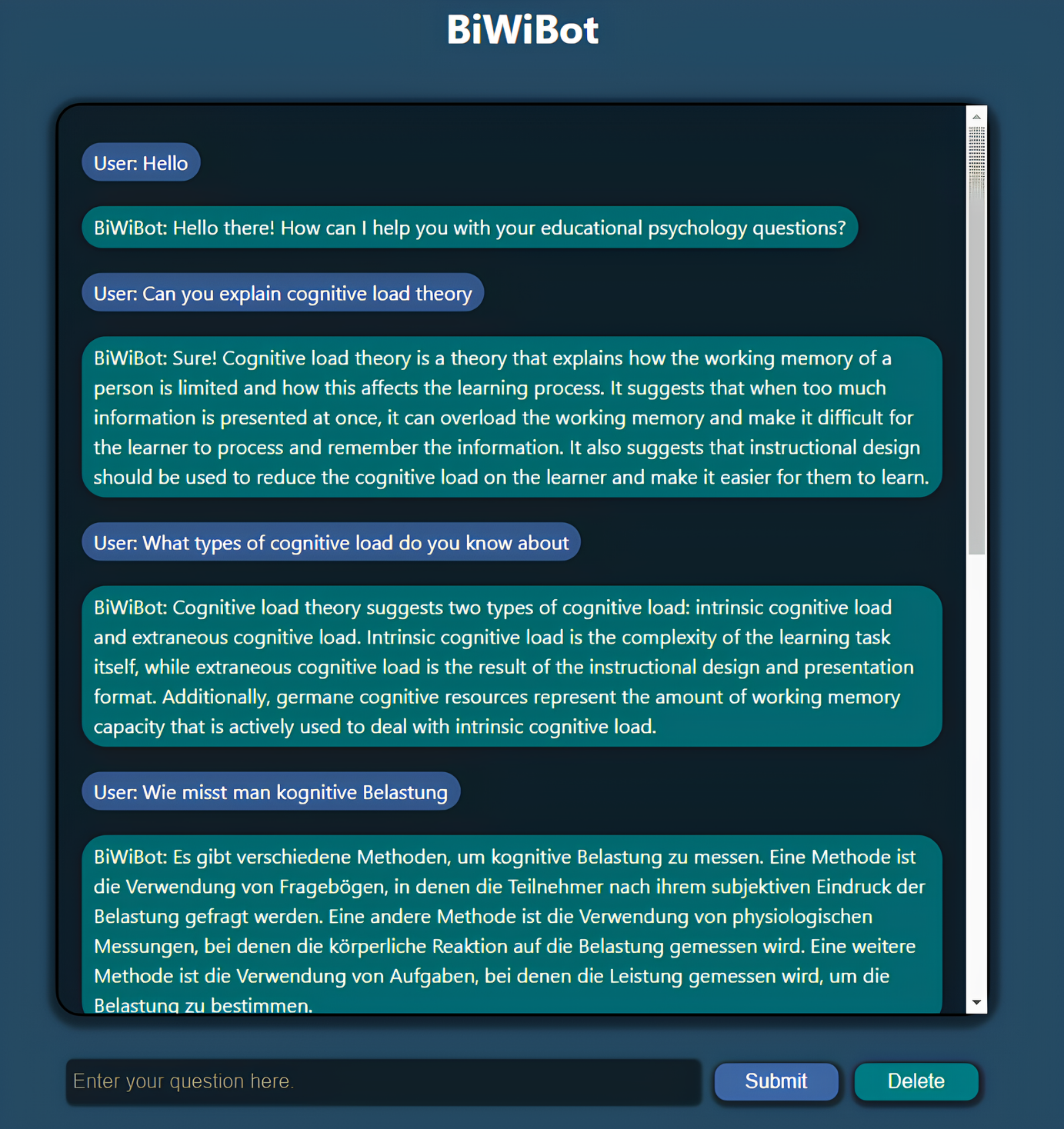


Figure 1 Proof of Concept (Version 1)


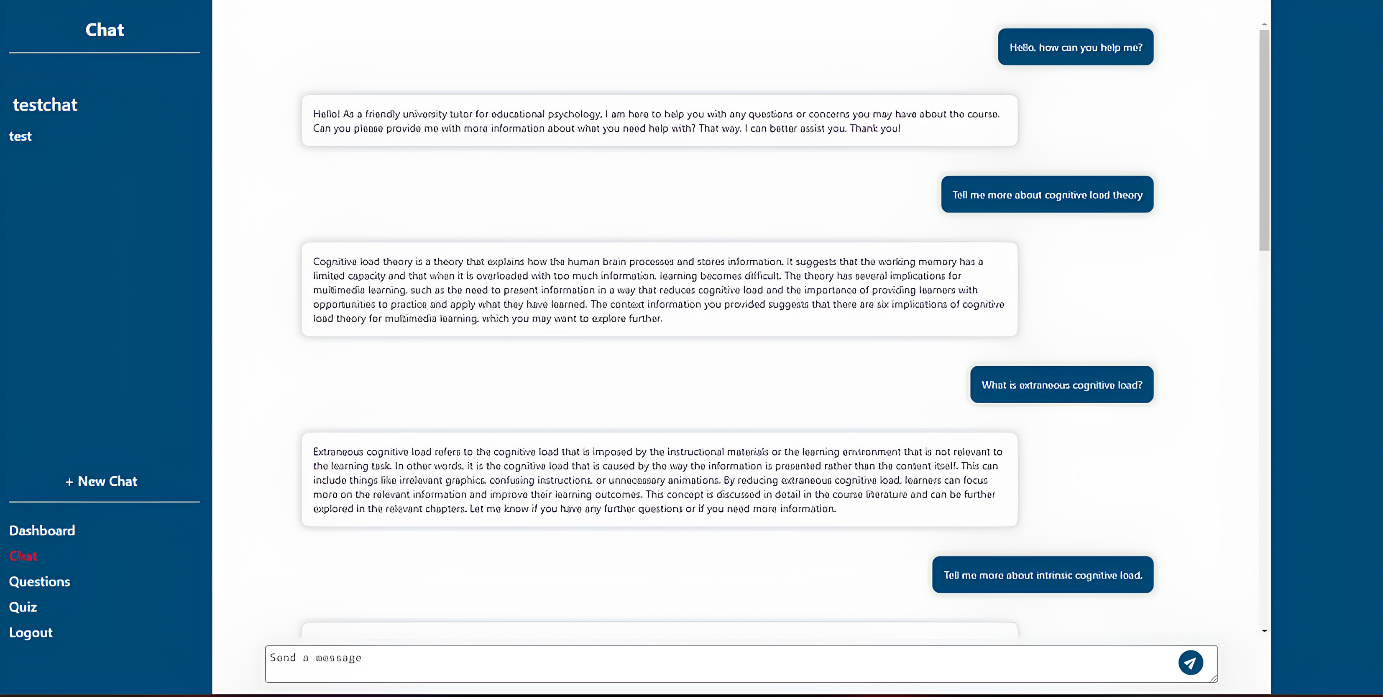


Figure 2 Prototype (Version 2)


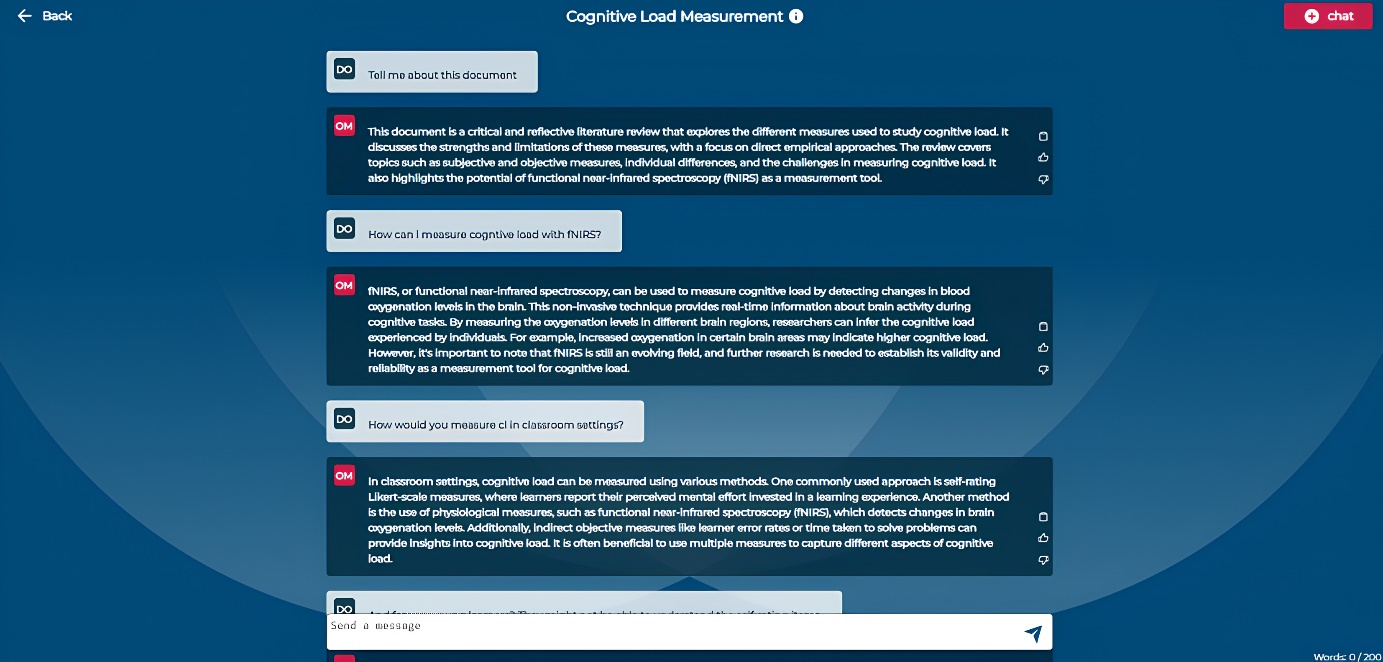


Figure 3 Final Version (Version 3)
